# Two specific interactions of GATE16 with TRPML3 and RAB33B regulate autophagy

**DOI:** 10.1038/s41598-025-16951-0

**Published:** 2025-08-25

**Authors:** Jiwoo Park, Areum Choi, Jin Kwon, Suzi Choi, Yun Min Park, Hyun Jin Kim

**Affiliations:** Department of Physiology, Sungkyunkwan University School of Medicine, Suwon, 16419 South Korea

**Keywords:** ATG8, Autophagy, GATE16, RAB33B, TRPML3, Cell biology, Molecular biology

## Abstract

ATG8s are essential for autophagy as they recruit various machinery to autophagic structures. We previously reported that the intracellular Ca^2+^ channel TRPML3 specifically interacts with the mammalian ATG8 homolog GATE16, but not LC3B to increase autophagy. However, the underlying mechanism and the role of this specific interaction remain unclear. Here, we report that single amino acid motifs in GATE16 and TRPML3 determine the specificity of this interaction and its function in autophagy. We also discovered that RAB33B, a Golgi-resident small GTPase, functionally interacts with TRPML3 in autophagy and contains an LC3-interacting region (LIR) motif. Surprisingly, RAB33B specifically interacted with GATE16, but not with other ATG8s through an LIR motif, and disrupting this LIR motif inhibited autophagy. Upon induction of autophagy, RAB33B was recruited from the Golgi to the phagophore in an LIR-dependent manner, enhancing the interaction between RAB33B and TRPML3 while promoting autophagy. These results suggest that specific interactions involving GATE16 play a crucial role in autophagy by recruiting TRPML3 and RAB33B, forming protein complexes at the phagophore to promote autophagosome formation.

**Supplementary Information:**

The online version contains supplementary material available at 10.1038/s41598-025-16951-0.

## Introduction

Autophagy is the process that allows the clearance of cytoplasmic cargoes isolated in autophagosomes and delivered to the lysosome for degradation^1–3^. In autophagy, autophagy-related (ATG) proteins play key roles in various stages of the process. For example, ATG8 proteins, widely used markers to study autophagosome biogenesis and trafficking^4^recruit proteins containing the LC3-interacting region (LIR) to the autophagy process^5^. In mammals, ATG8 proteins are divided into two subfamilies: LC3s (LC3A, LC3B, LC3C) and GABARAPs (GABARAP, GABARAPL1, GATE16)^6^.

The LIR motif plays a key role in recruiting autophagy receptors to LC3 and other ATG8 family proteins anchored in the phagophore membrane^7^. This interaction is governed by 15 to 20 amino acid sequences, which specifically bind to the LIR docking site of ATG8 proteins^8^. The LIR motif binds basically through an aromatic residue (W/Y/F) and a hydrophobic residue (L/I/V) fitting into two hydrophobic pockets created by the structure of ATG8 family proteins^9^. However, recent studies have shown that a lot of ATG8-interacting proteins are independent of canonical LIR interaction, suggesting alternative binding mechanisms^5,10^.

In general, LC3s preferentially bind to the LIR-containing proteins involved in autophagosome transport and selective autophagy process^5^. On the other hand, GABARAPs favorably bind to LIR-containing core autophagy components involved in autophagosome formation, such as ULK1, ULK2, ATG13, ATG14, and FIP200^11,12^. Although there are several reports on how selective binding to GABARAPs is achieved^13,14^the molecular determinants on the ATG8 side for the interactions between ATG8s and autophagy components remain elusive.

The intracellular Ca^2+^ channel TRPML3 is a downstream effector of PI3P, releasing Ca^2+^ from the phagophore to promote autophagosome biogenesis^15^. Our previous study revealed that TRPML3 specifically binds to GATE16, but not to LC3B^16^. However, the precise mechanisms underlying the specificity and role of the interaction need to be investigated.

In this study, we demonstrate that the specificity of the TRPML3-GATE16 interaction is determined by single amino acid motifs in both TRPML3 and GATE16. Furthermore, we show that this interaction plays a crucial role in autophagy by identifying another specific interaction of GATE16 with a core autophagy component via an LIR motif.

## Results

### The specific interaction between GATE16 and TRPML3 via Leu94 in GATE16 regulates autophagy

**Fig. 1:**
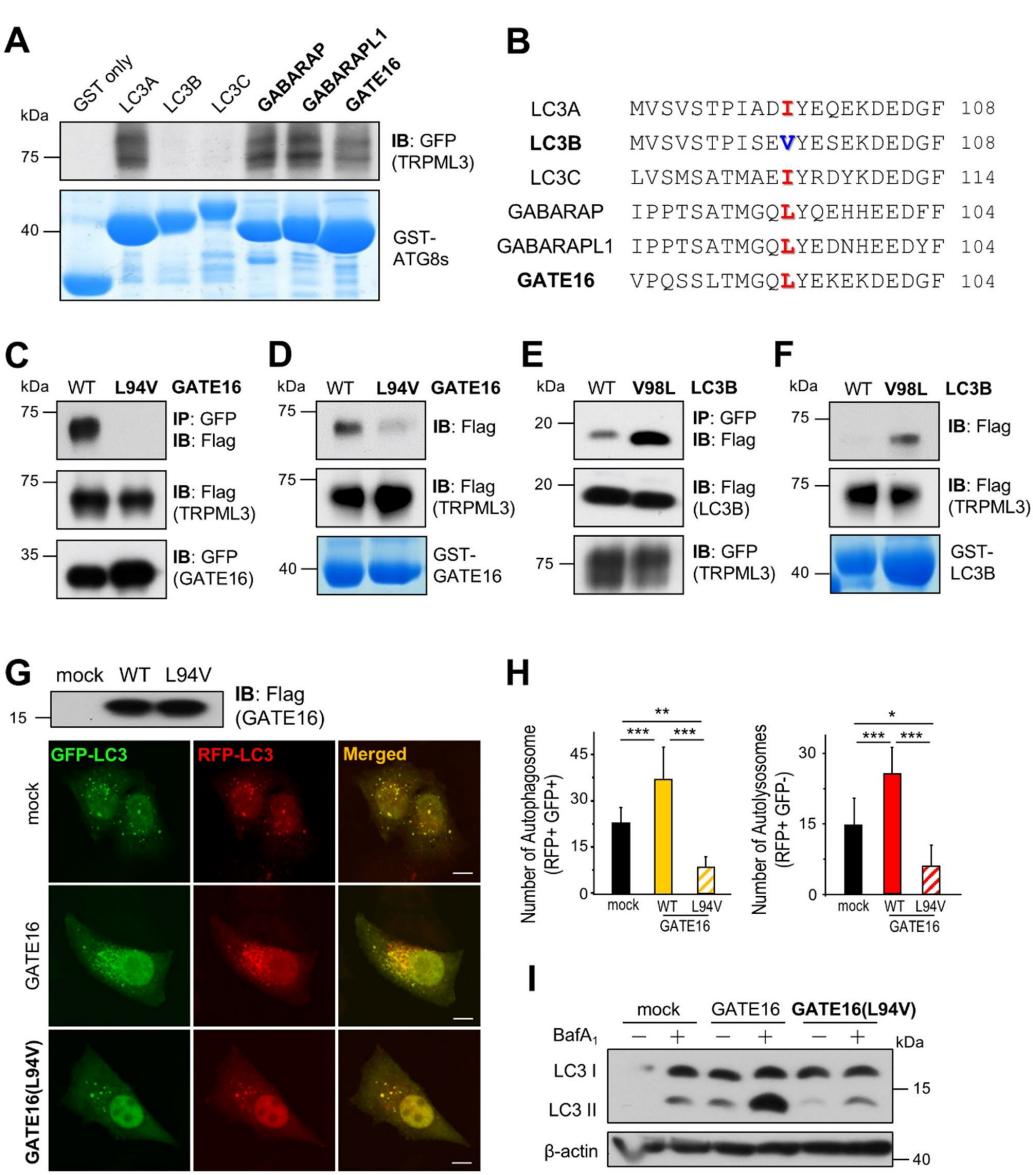
The specificity of the TRPML3-GATE16 interaction and its role in autophagy. (**A**) GST-tagged ATG8 homologs coupled to glutathione Sepharose beads were incubated with whole cell lysates from HEK293T cells expressing mock and GFP-TRPML3. Elutes were subjected to SDS-PAGE and immunoblotted with anti-GFP antibody. (**B**) Sequence alignment of mammalian ATG8 proteins. (**C**) Cell lysates from HEK293T cells expressing WT or L94V GFP-GATE16 with Flag-TRPML3 were subjected to immunoprecipitation with anti-GFP antibody and probed with anti-Flag antibody. (**D**) GST-GATE16 or GST-GATE16(L94V) coupled to glutathione Sepharose beads were incubated with whole cell lysates from HEK293T cells expressing Flag-TRPML3. Elutes were subjected to SDS-PAGE and immunoblotted with anti-Flag antibody. (**E**) Cell lysates of HEK293T cells expressing WT or Flag-LC3B(V98L) with GFP-TRPML3 were subjected to immunoprecipitation with anti-GFP antibody and were probed with anti-Flag antibody. (**F**) GST-LC3B or GST-LC3B(V98L) coupled to glutathione Sepharose beads were incubated with whole cell lysate from HEK293T cells expressing Flag-TRPML3. Elutes were subjected to SDS-PAGE and immunoblotted with anti-Flag antibody. (**G**) HeLa cells expressing mock, Flag-GATE16, or Flag-GATE16(L94V) were subjected to western blot analysis using an anti-Flag antibody (top), and cells expressing tfLC3 along with these constructs were serum-starved for 2 h and analyzed by confocal microscopy (bottom). (**H**) The number of autophagosomes and autolysosomes in panel (G) were quantified and presented as mean ± SD of 13–15 cells (*p < 0.05, **p < 0.001, ***p < 0.005, ANOVA). (**I**) HEK293T cells expressing mock, GATE16, GATE16(L94V) were treated with vehicle or 100 nM bafilomycin A_1_ in serum-free medium for 2 h and processed for western blot analysis to assay endogenous LC3 levels. β-actin was used as a loading control. BafA_1_, bafilomycin A_1_.

Our previous study showed that the intracellular Ca^2+^ channel TRPML3 specifically interacts with GATE16, but not with LC3B^16^. To understand the relationship between TRPML3 and ATG8 family proteins, we investigated the interaction of TRPML3 with ATG8 homologs using a GST pull-down assay. As shown in Fig. 1A, TRPML3 bound to LC3A, GABARAP, GABARAPL1, and GATE16. We observed almost complete overlaps between TRPML3 and all ATG8 homologs in confocal images (Supplementary Fig. 1A). However, the TRPML3-GCaMP6 signal, which reflects a TRPML3-mediated Ca^2+^ signal^15^, only overlapped with LC3C and GABARAP subfamily members (Supplementary Fig. 1B), suggesting that GABARAPs functionally interact with TRPML3. To clarify the binding specificity between LC3s and GABARAPs, we searched for potential LIR motifs using the iLIR database and manual inspection of the TRPML3 amino acid sequence, but found none. Thus, we sought to identify the interaction site on the ATG8 side by performing a sequence alignment of ATG8 homologs. Interestingly, only LC3B contains a valine at a specific site (Val98 in LC3B), whereas the other LC3s and GABARAPs have (iso)leucine at the same site (Leu94 in GATE16) (Fig. 1B). This result suggests that the specific site may be important for the interaction between TRPML3 and ATG8s. To determine the role of this site in the TRPML3-GATE16 interaction, we mutated Leu94 in GATE16 to Valine and performed co-immunoprecipitation (co-IP) and GST pull-down assays. As shown in Fig. 1C and D, the TRPML3-GATE16 interaction was severely inhibited by the L94V mutation. On the contrary, the TRPML3-LC3B interaction was rescued by the V98L mutation in LC3B (Fig. 1E and F), suggesting that the Leu94 in GABARAPs is crucial for the interaction with TRPML3. As the TRPML3-GATE16 interaction regulates autophagy^16^we investigated the effect of the identified site on autophagy. We determined the number of autophagosomes and autolysosomes using mRFP-GFP tandem fluorescent-tagged LC3 (tfLC3). Overexpression of GATE16 significantly increased the yellow (autophagosome) and red (autolysosome) particles, indicating an increase in autophagy flux. However, GATE16(L94V), which cannot bind to TRPML3, showed a significant decrease in both yellow and red particles, indicating that autophagosome formation is defective in both basal (Supplementary Fig. 2A and B) and autophagy-induced conditions (Fig. 1G and H). These results were double-checked with an autophagic flux assay (Supplementary Fig. 2C and Fig. 1I). All these data demonstrate that a single amino acid motif, Leu94 in GATE16, is crucial for the interaction with TRPML3, which regulates autophagy.

### Cys50 in TRPML3 is responsible for the TRPML3-GATE16 interaction and the subsequent increase in autophagy

**Fig. 2:**
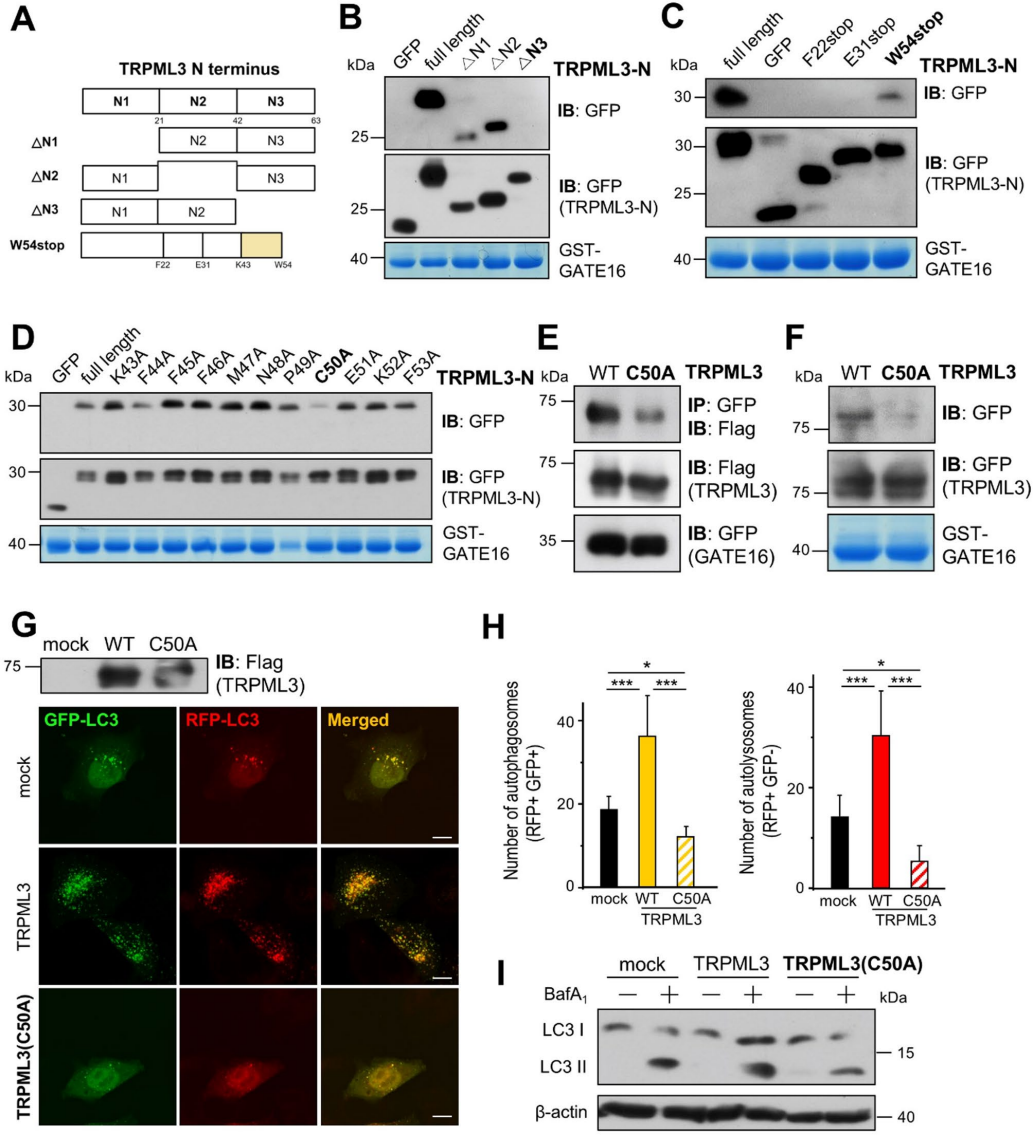
Effect of the C50A mutation in TRPML3 on the TRPML3-GATE16 interaction and autophagic flux. (**A**) Schematic of TRPML3-N terminus. (**B**,**C**,**D**) GST-GATE16 coupled to glutathione Sepharose beads were incubated with whole cell lysates from HEK293T cells expressing either mock or the indicated GFP-TRPML3-N proteins. Elutes were subjected to SDS-PAGE and immunoblotted with anti-GFP antibody. (**E**) Cell lysates of HEK293T cells expressing WT or Flag-TRPML3(C50A) with GFP-GATE16 were subjected to immunoprecipitation with anti-GFP antibody and were probed with anti-Flag antibody. (**F**) GST-GATE16 coupled to glutathione Sepharose beads were incubated with whole cell lysate from HEK293T cells expressing WT or GFP-TRPML3(C50A). Elutes were subjected to SDS-PAGE and immunoblotted with anti-GFP antibody. (**G**) HeLa cells expressing mock, Flag-TRPML3, or Flag-TRPML3(C50A) were subjected to western blot analysis using an anti-Flag antibody (top), and cells expressing tfLC3 along with these constructs were serum-starved for 2 h and analyzed by confocal microscopy (bottom). (**H**) The number of autophagosomes and autolysosomes in panel (G) were quantified and presented as mean ± SD of 12–13 cells (*p < 0.05, ***p < 0.005, ANOVA). (**I**) HEK293T cells expressing mock or TRPML3 or TRPML3(C50A) were treated with vehicle or 100 nM bafilomycin A_1_ in serum-free medium for 2 h and processed for western blot analysis to assay endogenous LC3 levels. β-actin was used as a loading control.

Next, to identify the interaction site on the TRPML3 side, we generated several deletion and truncation mutants of the TRPML3-N terminus (Fig. 2A). We narrowed down the expected binding site using a GST pull-down assay and noticed that Cys50 in TRPML3 may be the binding site for GATE16 (Fig. 2B-D). Co-IP (Fig. 2E) and GST pull-down assays (Fig. 2F) revealed that the TRPML3-GATE16 interaction is greatly inhibited by C50A mutation, confirming that Cys50 in TRPML3 is important for the TRPML3-GATE16 interaction. We then checked the effect of the mutation on autophagy using tfLC3 and autophagic flux assay, which showed a marked decrease in autophagy in both fed (Supplementary Fig. 3A-C) and starved conditions (Fig. 2G-I). Together, these results suggest that a single amino acid motif Cys50 in TRPML3 is responsible for the TRPML3-GATE16 interaction and subsequent increase in autophagy.

### TRPML3 functionally interacts with RAB33B, which regulates autophagy

**Fig. 3:**
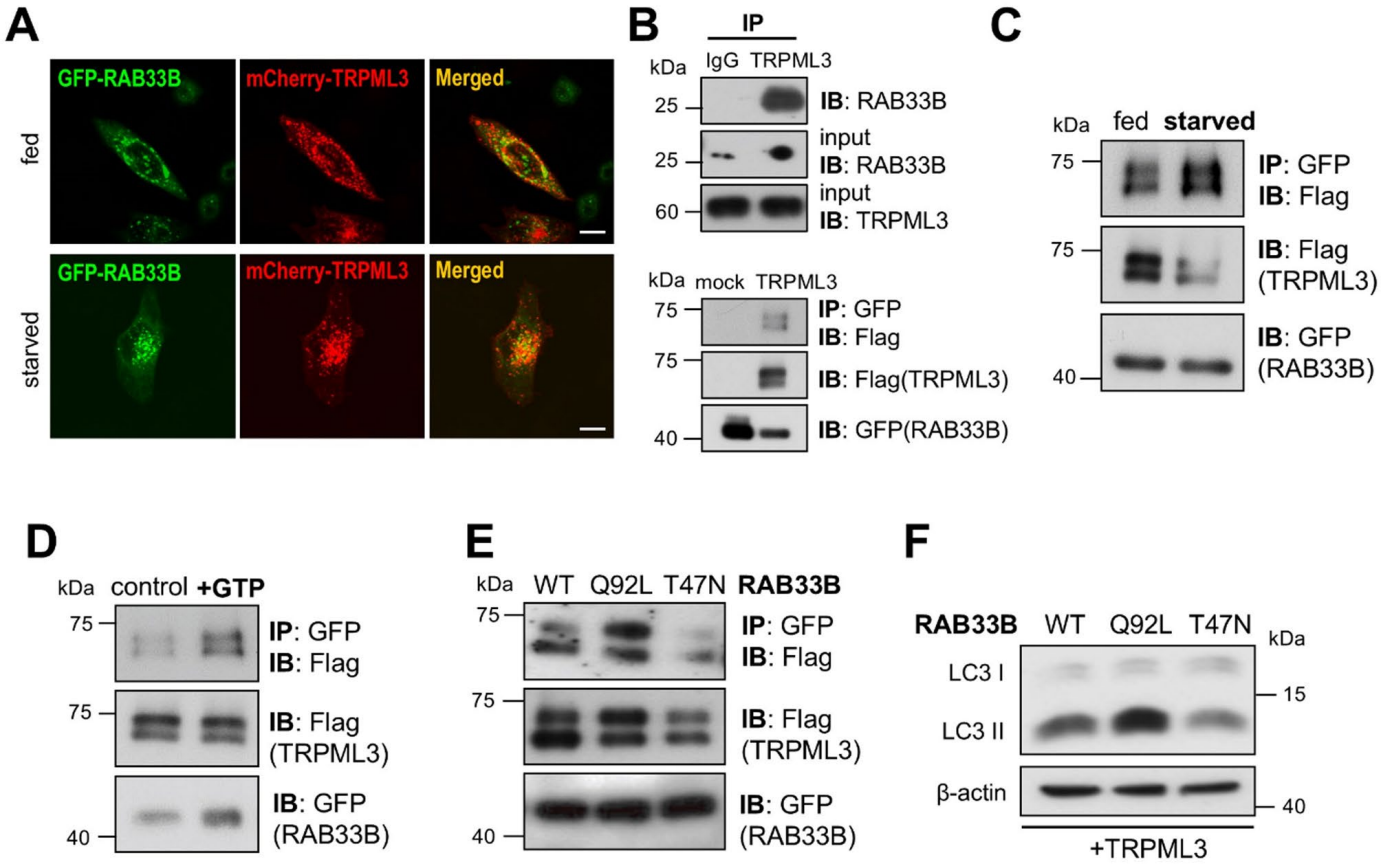
Relationship between RAB33B and TRPML3 in autophagy. (**A**) HeLa cells expressing GFP-RAB33B and mCherry-TRPML3 were kept in fed media or serum-starved for 2 h, followed by confocal microscopy analysis. (**B**) Cell lysates from HEK293T cells were subjected to immunoprecipitation with control IgG or anti-TRPML3 antibody and probed with anti-RAB33B antibody (top). Cell lysates of HEK293T cells expressing GFP-RAB33B with Flag-TRPML3 were subjected to immunoprecipitation with anti-GFP antibody and were probed with anti-Flag antibody (bottom). (**C**) This analysis is similar to (B), except cells were kept in full media or serum-starved for 2 h. (**D**) This analysis is similar to (**B**), except cells were treated with vehicle or 0.5 mM GTP. (**E**) Cell lysates of HEK293T cells expressing WT, Q92L, T47N GFP-RAB33B with Flag-TRPML3 were subjected to immunoprecipitation with anti-GFP antibody and probed with anti-Flag antibody. (**F**) HEK293T cells expressing WT, Q92L, T47N GFP-RAB33B with Flag-TRPML3 were processed for western blot analysis to assay endogenous LC3 levels. β-actin was used as a loading control.

In further investigating the role of the TRPML3-GATE16 interaction, we focused on RAB33B which was screened as an interacting protein of TRPML3 in our previous study^16^. RAB33B is a Golgi-resident RAS-associated binding protein closely linked to autophagy^17^. RAB33B directly interacts with ATG16L1^18^ and is essential for recruiting the ATG16L1 complex to the phagophore during starvation-induced autophagy^19^. We confirmed the interaction between TRPML3 and RAB33B using both endogenous and overexpression-based co-IP (Fig. 3B). The interaction was increased by starvation (Fig. 3C), as evidenced by the results in Fig. 3A showing the increased overlap in starved conditions. RAB33B is a small GTP-binding protein^20^ that is activated by GTP and inactivated by GDP^21^. Notably, the TRPML3-RAB33B interaction was increased by GTP treatment (Fig. 3D) and GTP-locked active mutation (Q92L) but decreased by GDP-locked inactive mutation (T47N) (Fig. 3E). Moreover, we also observed that the lipidation of LC3 is increased by RAB33B(Q92L) but decreased by RAB33B(T47N) in the presence of TRPML3 (Fig. 3F). All these data indicate that TRPML3 interacts with an active form of RAB33B to be an effector protein upon autophagy induction.

### RAB33B specifically interacts with GATE16 via the LIR motif in autophagy

**Fig. 4:**
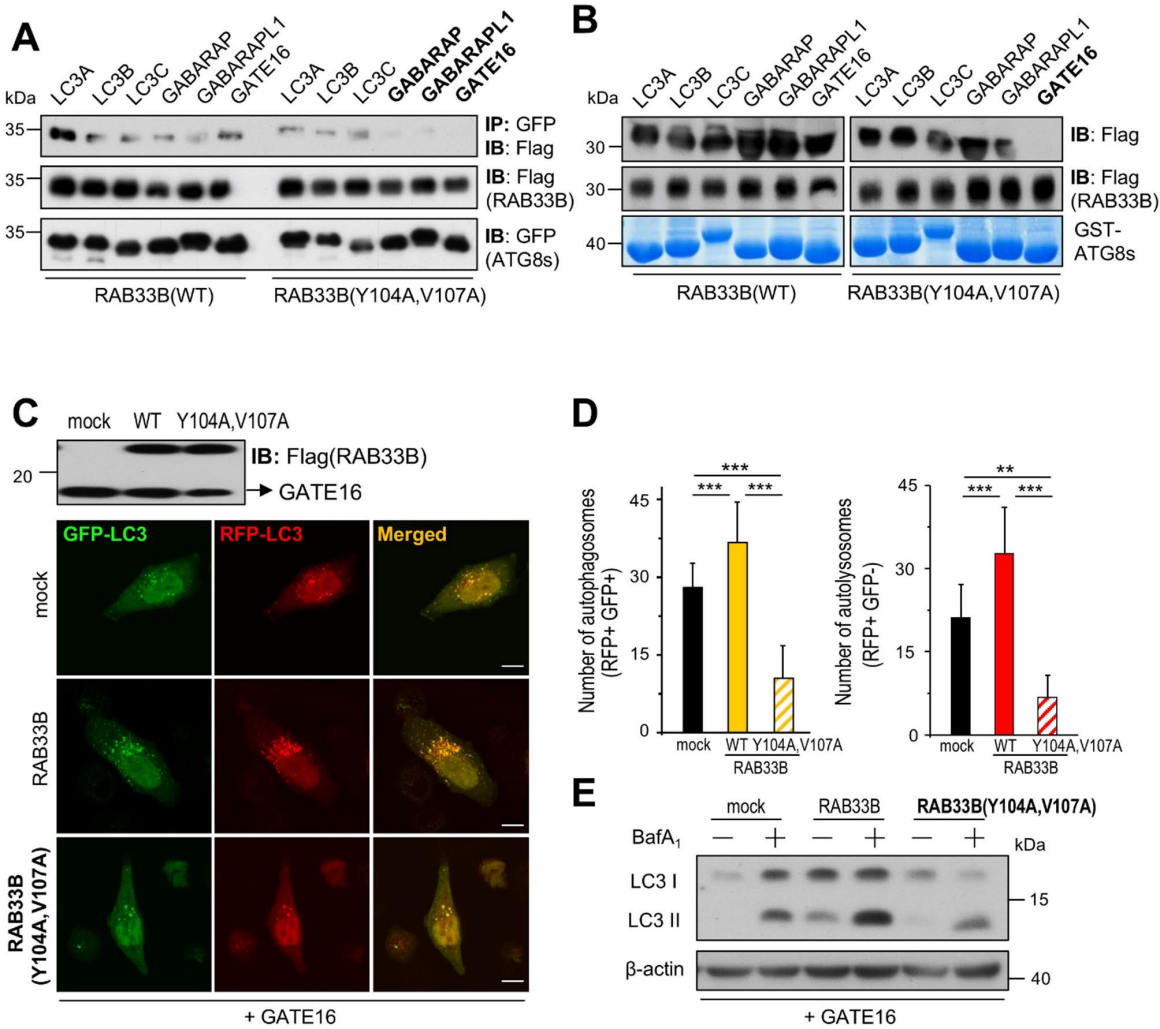
The specific interaction of GATE16-RAB33B via LIR and its role in autophagy. (**A**) Cell lysates of HEK293T cells expressing Flag-RAB33B or Flag-RAB33B(Y104A, V107A) with GFP-tagged ATG8 homologs were subjected to immunoprecipitation with anti-GFP antibody and probed with anti-Flag antibody. (**B**) GST-tagged ATG8 homologs coupled to glutathione Sepharose beads were incubated with whole cell lysate from HEK293T cells expressing Flag-RAB33B or Flag-RAB33B(Y104A, V107A). Elutes were subjected to SDS-PAGE and immunoblotted with anti-Flag antibody. (**C**) HeLa cells expressing Flag-GATE16 with mock, Flag-RAB33B, or Flag-RAB33B(Y104A, V107A) were subjected to western blot analysis using an anti-Flag antibody (top), and cells expressing tfLC3 along with these constructs were serum-starved for 2 h and analyzed by confocal microscopy (bottom). (**D**) The number of autophagosomes and autolysosomes in panel (C) were quantified and presented as mean ± SD of 11–16 cells (**p < 0.01, ***p < 0.005, ANOVA). (**E**) HEK293T cells expressing mock, RAB33B, RAB33B(Y104A, V107A) were treated with vehicle or 100 nM bafilomycin A_1_ in serum-free medium for 2 h and processed for western blot analysis to assay endogenous LC3 levels. β-actin was used as a loading control.

RAB33B is known to translocate from the Golgi to the phagophore upon autophagy induction^18^. Since ATG8 proteins recruit core autophagy components to the autophagic structures^22^we hypothesized that ATG8 proteins may recruit RAB33B upon autophagy induction. Considering that this process is usually mediated via an LIR motif^5^we searched for potential candidates of LIR motifs in RAB33B using the iLIR database and manual inspection of the RAB33B amino acid sequence (Supplementary Fig. 4A). Among the four candidate LIR motifs, LIR3 was excluded due to undetectable expression of its mutant. Given our previous focus on GATE16 among the ATG8 proteins, we assessed its interaction with the candidate LIR motif mutants. Notably, the LIR2 mutant, hereafter referred to as RAB33B(Y104A, V107A), exhibited a markedly reduced interaction (Supplementary Fig. 4B). We additionally examined the interaction between LIR1 and LIR4 mutants with ATG8 family proteins (Supplementary Fig. 4C). These LIR mutants showed detectable binding primarily with GABARAP subfamily proteins. In contrast, co-IP experiments revealed that RAB33B(Y104A, V107A) is unable to bind to GABARAPs (Fig. 4A). Surprisingly, the results showed that the RAB33B(Y104A, V107A) does not bind to only GATE16 among GABARAPs (Fig. 4B), suggesting that GATE16 is responsible for RAB33B recruitment via this LIR motif in autophagy. Furthermore, RAB33B(Y104A, V107A) significantly inhibited autophagosome formation even in the presence of GATE16 under both fed (Supplementary Fig. 5A-C) and starved conditions (Fig. 4C-E). These results point out that RAB33B specifically interacts with GATE16 through the LIR motif in RAB33B and this interaction is crucial for autophagosome formation.

### The specific GATE16-RAB33B interaction via the LIR motif mediates RAB33B recruitment to the phagophore upon induction of autophagy

**Fig. 5:**
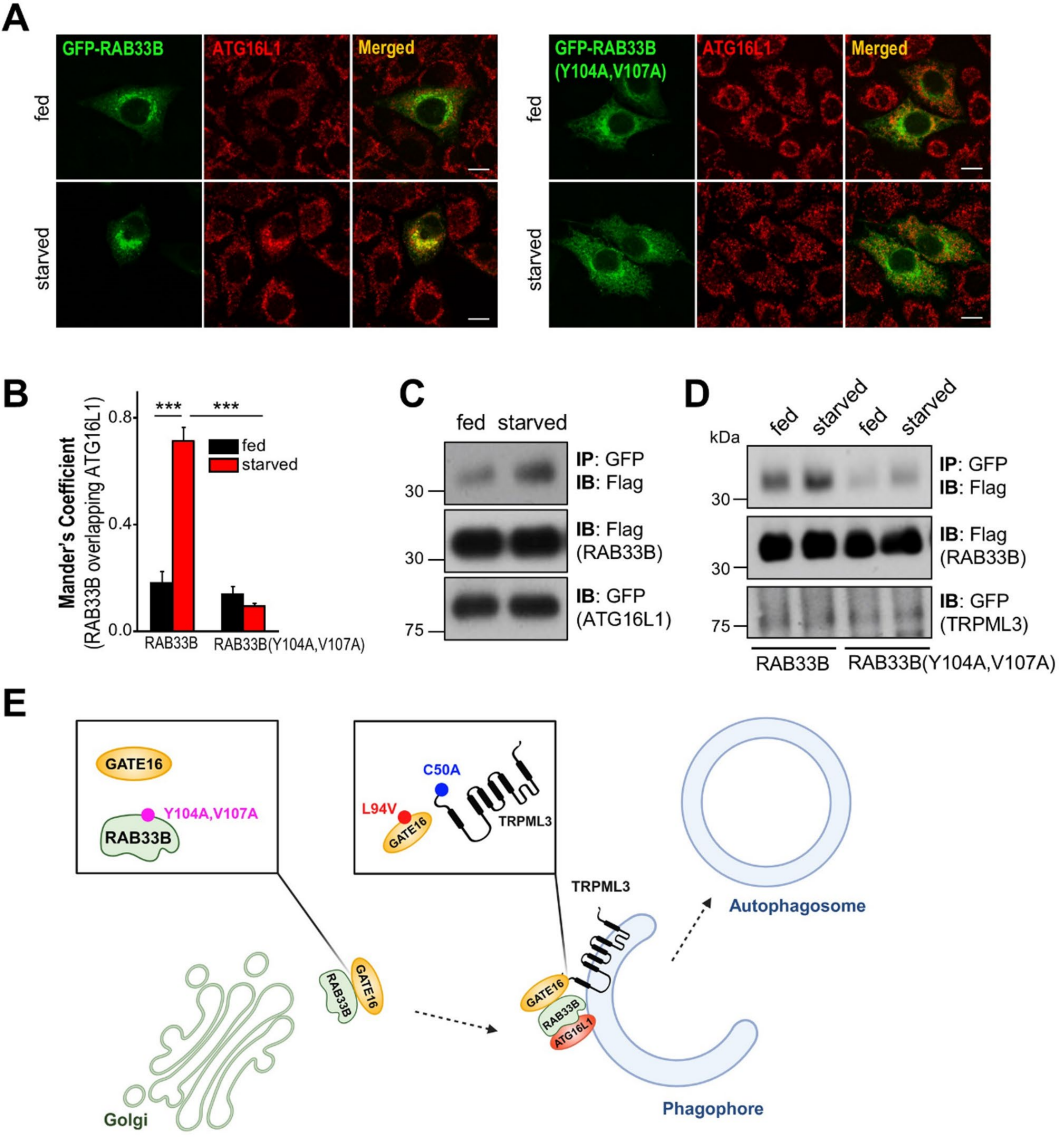
Role of the RAB33B-GATE16 interaction in phagophore recruitment and RAB33B-TRPML3 interaction. (**A**) HeLa cells expressing WT or Y104A, V107A GFP-RAB33B were stained with anti-ATG16L1, were kept in fed media or serum-starved for 2 h followed by confocal microscopy analysis. (**B**) The overlap in each condition was determined using ImageJ software and is presented as mean ± SEM of 3–4 cells (***p < 0.005, Student’s *t*-test). (**C**) Cell lysates of HEK293T cells expressing GFP-ATG16L1 with Flag-RAB33B were kept in fed media or serum-starved 2 h, subjected to immunoprecipitation with anti-GFP antibody and were probed with anti-Flag antibody. (**D**) Cell lysates of HEK293T cells expressing Flag-RAB33B or Flag-RAB33B(Y104A, V107A) with GFP-TRPML3 were kept in fed media or serum-starved 2 h, subjected to immunoprecipitation with anti-GFP antibody and probed with anti-Flag antibody. (**E**) Model depicting the relationships between TRPML3, GATE16 and RAB33B in autophagy.

To find the role of the interaction between RAB33B and GATE16, we examined whether this interaction affects the subcellular localization of RAB33B. We observed that RAB33B is recruited from the Golgi to the phagophore, indicative of increased overlap with ATG16L1 under starved conditions. However, the overlap between RAB33B(Y104A, V107A) and ATG16L1 significantly decreased even in starved conditions (Fig. 5A and B). The results demonstrate that the RAB33B-GATE16 interaction via the LIR motif is crucial for RAB33B recruitment in autophagy. Consistent with this observation, the interaction between RAB33B and ATG16L1 is enhanced under starvation conditions, suggesting that RAB33B is recruited to phagophores during autophagy induction (Fig. 5C). Our previous study showed that TRPML3 also translocates to newly forming autophagosomes upon induction of autophagy^23^. Therefore, we examined the interaction between TRPML3 and RAB33B using the RAB33B LIR mutant. Indeed, the LIR mutation in RAB33B almost completely abrogated the TRPML3-RAB33B interaction even in starved conditions, indicating that their interaction occurs at the phagophore (Fig. 5D). Collectively, all these data suggest that the specific interaction between GATE16 and RAB33B is essential for forming a complex with TRPML3 at the phagophore in autophagosome formation (Fig. 5E).

## Discussion

ATG proteins control dynamic membrane events during autophagy^24^. Among them, ATG8s divided into two subfamilies, LC3 and GABARAP, play important roles in biogenesis and maturation of autophagosomes^25,26^. They have common roles while performing different roles, with subfamily-specific binding to LIR-containing autophagy proteins^17,27^. In this study, we aimed to solve the binding specificity of TRPML3 to GATE16^16^. However, we could not apply the previously known principles since TRPML3 has no expected LIR motifs. Instead, we took a different approach and identified the molecular determinants on the ATG8s side. Figure 1 shows that the Leu94 residue in GATE16 and other GABARAP subfamily members are responsible for TRPML3-ATG8 interactions and autophagy. Surprisingly, LC3B, which normally does not bind to TRPML3, can interact with it when a Val-to-Leu mutation is introduced at the same site, highlighting the critical role of the Leu residue. We also tried to determine the binding site of TRPML3 to GATE16 and found that Cys50 in TRPML3 is an important residue for the TRPML3-GATE16 interaction (Fig. 2). However, this residue does not match any known ATG8 binding sites, including non-canonical LIR motifs^28^. Although we were unable to confirm all protein–protein interactions at the endogenous level to fully establish their physiological relevance, future validation using endogenous proteins will be essential to further support these findings.

The intracellular Ca^2+^ channel TRPML3 is a downstream target of PI3P, supplying Ca^2+^ to the phagophore microdomain to trigger membrane fusion in autophagosome formation^15^. Besides TRPML3, various factors such as SNAREs, tethers, and RAB GTPases must cooperate to facilitate autophagic membrane fusion. RAB33B is known to participate in autophagosome formation^29,30^however, the mechanism by which RAB33B is recruited and plays a role in autophagy remained unclear. Here, we show that RAB33B is an LIR-containing protein that binds specifically to GATE16 via LIR motif but not other ATG8 family members (Fig. 4), revealing a subfamily-specific interaction. This specificity likely enables recruitment of RAB33B to the phagophore, where it cooperates with TRPML3 to promote membrane fusion and autophagosome biogenesis (Fig. 5).

Our findings raise the possibility that other autophagy-related proteins may also utilize atypical interfaces or exhibit selective ATG8 subfamily preferences, suggesting a broader and diverse ATG8 interaction landscape than previously understood. Future investigations into the structural basis and regulatory mechanisms of these non-canonical interactions will be important for understanding autophagy’s complexity. While our findings provide insights into the molecular mechanisms involving ATG8 proteins in autophagy, they are based on in vitro experiments; therefore, further in vivo studies will be necessary to validate these interactions and to clarify their deeper physiological significance.

In summary, our study demonstrates that GATE16 recruits RAB33B and its effector TRPML3 to the phagophore through specific interactions, forming a fusion complex essential for autophagosome formation.

## Materials and methods

### Plasmid construction and mutagenesis

pEGFPC1-TRPML3, p3XFLAG-CMV-7.1-TRPML3 and p3XFLAG-CMV-7.1-mCherry-TRPML3 were prepared as previously detailed^16,31^. Plasmids containing human *LC3A*, *LC3C*, *GABARAPL1*, and *RAB33B* were amplified by PCR and cloned into pEGFPC1, p3XFLAG-CMV-7.1, or pGEX-4T1 using EcoRI and SalI sites. Plasmids containing the human *GABARAP* were amplified by PCR and cloned into pEGFPC1, p3XFLAG-CMV-7.1 and pGEX-4T1 using BglII and SalI sites. pEGFP-LC3 was a gift from Tamotsu Yoshimori (Addgene, 21073; http://n2t.net/addgene:21073; RRID: Addgene_21073). p3XFLAG-CMV-7.1-LC3 was generated as described previously^15^. Plasmids that contain *GATE16* were generated as detailed previously^16^. ptfLC3 was a gift from Tamotsu Yoshimori (Addgene, 21074; http://n2t.net/addgene:21074; RRID: Addgene_21074). The coding sequence of the TRPML3-N terminus (aa 31–66) was fused into pEGFPC1 using EcoRI and SalI sites. All deletion, truncation, or point mutants were generated by site-directed mutagenesis. All mutations were confirmed by sequencing the entire DNA insert to verify the presence of the desired mutation and the absence of extraneous mutations. GFP-ATG16L1 was generated by subcloning ATG16L1 from p3xFLAG-CMV10-mApg16L (p3xFLAG-CMV10-mApg16L was a gift from Noboru Mizushima (Addgene plasmid # 24302; http://n2t.net/addgene:24302; RRID: Addgene_24302)).

### Antibodies and reagents

The following antibodies were used: anti-GFP (Invitrogen, A11122), HRP-FLAG (Sigma, A8592), anti-LC3B (Thermo Fisher Scientific, PA1-16930), HRP-conjugated β-actin (Sigma, A3854), control IgG (Sigma, 12–370), anti-TRPML3 (Alomone Labs, ACC-083), anti-RAB33B (Santa Cruz, sc-81920), and anti-ATG16L1 (Abcam, ab187671). Bafilomycin A_1_ (Merck Millipore, 196000) was used for autophagy assay.

### Cell culture and transfection

HEK293T or HeLa cells were maintained in DMEM (Sigma, D5796) supplemented with 10% fetal bovine serum (FBS; Gibco, 16000-044) and 1% antibiotics (Gibco, 15240062) in a humidified incubator at 37 °C and 5% CO_2_. Cells were plated one day before transfection and transfected with plasmid DNA using Lipofectamine 2000 (Invitrogen, 11668019), according to the manufacturer’s instructions.

### Confocal microscopy and immunocytochemistry

HeLa cells transfected with the indicated constructs were grown on glass coverslips, fixed with 4% paraformaldehyde, and permeabilized by incubation with 0.05% Triton X-100 at room temperature for 20 min. After fixation, nonspecific sites were blocked with 5% bovine serum albumin. Some cells were stained with a primary antibody overnight at 4 °C and incubated with a fluorescent secondary antibody for 1 h at RT. Coverslips were mounted on glass slides and analyzed using a Zeiss LSM 710 confocal microscope. The images were analyzed offline using NIH ImageJ™ software (National Institutes of Health).

### Western blotting

Protein samples were separated by SDS-PAGE and transferred to a hydrophobic polyvinylidene difluoride (PVDF) membrane. Membranes were blocked in 5% skim milk in Tween Tris-Buffered Saline (TTBS; pH 7.4, 25 mM Tris, 3 mM KCl, 140 mM NaCl, 0.1% Tween 20) for 1 h at RT and then incubated in specified antibodies for 18 h at 4 °C. After three washes, membranes were incubated for 2 h at RT with HRP-conjugated secondary antibody and HRP-conjugated β-actin antibody (1:10000; Sigma, A3854). Blots were developed using an ECL reagent (GE Healthcare, RPN2232).

### Co-immunoprecipitation

Transfected HEK293T cells were collected and lysed using a lysis buffer (50 mM HEPES, 150 mM NaCl, 2 mM MgCl_2_, 1 mM NaF, 1 mM Na_3_VO_4_, 1 tablet of protease inhibitor cocktail and with 1% Triton X-100 for whole cell lysate). The lysate was sonicated and any insoluble material was discarded by centrifugation at 14,000 rpm for 20 min. For co-immunoprecipitation, a specific antibody was added to each whole cell lysate and rotated for 30 min at 4 °C. Then, 20 µl of a 50% slurry of protein A agarose beads added to the lysate-antibody mixture and further rotated for an additional 2 h at 4 °C. The beads were washed five times with 0.5% Triton X-100 in PBS, eluted, and analyzed by immunoblotting. For co-immunoprecipitation with GTP treatment, vehicle or 0.5 mM GTP was added to the lysate before antibody addition, and the mixture was incubated on ice for 1 h. After the incubation, 10 mM MgCl_2_ was added.

### GST pull-down assay

GST-fusion proteins were expressed in the BL21-*Escherichia coli*-strain. Fusion protein production was induced by adding 100 nM Isopropyl β-D-1-thiogalactopyranoside to bacterial cultures grown to an A600 of 0.6. Bacteria were lysed by three cycles of rapid freezing/thawing and the insoluble material was removed by centrifugation at 14,000 rpm for 10 min. The supernatant was incubated with glutathione-Sepharose beads (Amersham Biosciences) for 1 h at 4 °C. Cell lysates from HEK293T cells expressing the indicated proteins were then incubated with the GST-fusion protein-loaded glutathione-Sepharose for 12 h at 4 °C. The beads were harvested by centrifugation and washed five times with PBS. Bound proteins were eluted with 5× sample buffer and analyzed by immunoblotting.

### Statistical analysis

Results are presented as mean ± standard deviation (SD) or mean ± standard error of the mean (SEM), as specified. Data were analyzed using Origin version 7.0. Depending on the experimental design, either Student’s *t*-tests or one-way ANOVA followed by Tukey’s post-hoc test were performed. A p-value < 0.05 was considered statistically significant. Experiments were repeated as indicated, and the number of biological replicates (n) is provided in the respective figure legends.

## Supplementary Information

Below is the link to the electronic supplementary material.

Supplementary Material 1

Supplementary Material 2

## Data Availability

All study data are included in the article and/or its supplementary information files.
