# Supplementary material for: Two specific interactions of GATE16 with TRPML3 and RAB33B regulate autophagy
Source: Sci Rep. 2025 Aug 25;15:31244. doi: 10.1038/s41598-025-16951-0 (PMC12378212; doi:10.1038/s41598-025-16951-0)
Supplement: Supplementary file 1 — Supplementary Material 1 [file 41598_2025_16951_MOESM1_ESM.pdf]

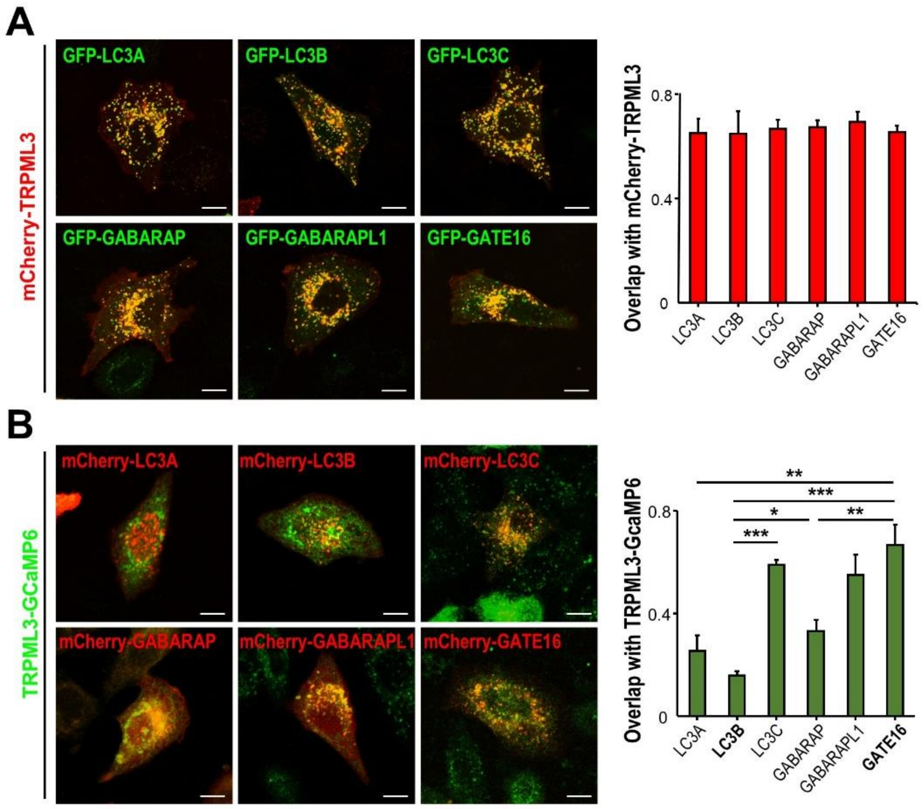

Supplementary Figure 1. Colocalization between ATG8 proteins and TRPML3. (**A**) HeLa cells co-expressing mCherry-TRPML3 with GFP-ATG8s were imaged by confocal microscopy and the overlaps were determined with ImageJ and given as mean ± SEM of 3-5 cells. (**B**) HeLa cells co-expressing TRPML3-GCaMP6 with mCherry-ATG8s were imaged by confocal microscopy and the overlaps were determined with ImageJ and given as mean ± SEM of 3-5 cells (*$p < 0.05$, **$p < 0.01$, ***$p < 0.005$, Student's *t*-test).

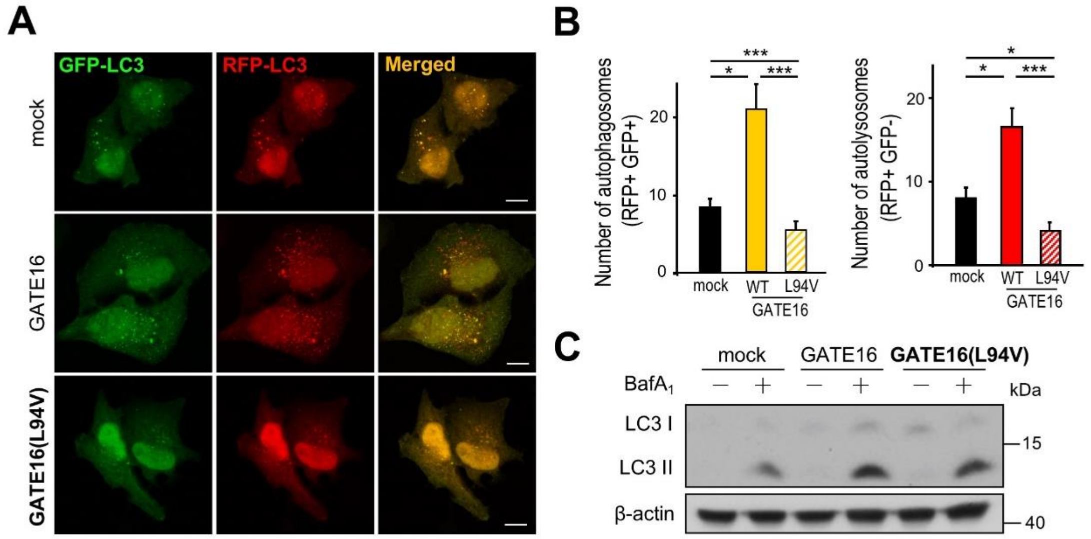

Supplementary Figure 2. Effects of GATE16 and GATE16(L94V) on autophagy flux under fed conditions. (**A**) HeLa cells expressing tfLC3 with mock, Flag-GATE16, Flag-GATE16(L94V) were analyzed by confocal microscopy. (**B**) The number of autophagosomes and autolysosomes in panel (G) were counted and presented as mean ± SEM of 3-5 cells (*p < 0.05, ***p < 0.005, Student's *t*-test). (**C**) HEK293T cells expressing mock, GATE16, GATE16(L94V) were treated with 100 nM bafilomycin A_1 for 4 h and processed for western blot analysis to assay endogenous LC3 levels. β-actin was used as a loading control. BafA_1, bafilomycin A_1

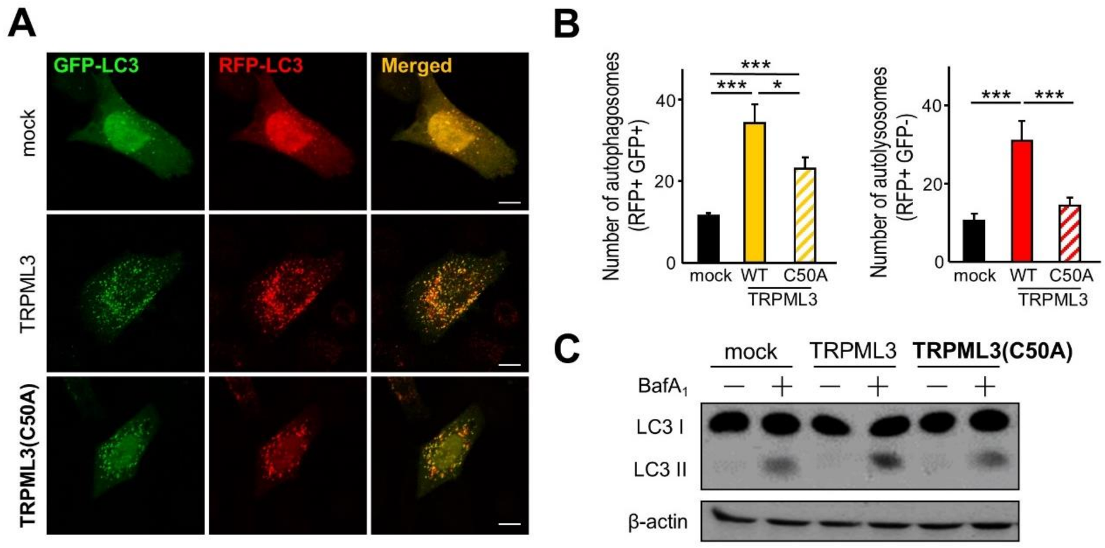

Supplementary Figure 3. Effects of TRPML3 and TRPML3(C50A) on autophagy flux under fed conditions. (**A**) HeLa cells expressing tfLC3 with mock, Flag-TRPML3, Flag-TRPML3(C50A) were analyzed by confocal microscopy. (**B**) The number of autophagosomes and autolysosomes in panel (G) were counted and presented as mean ± SEM of 3-5 cells (*$p < 0.05$, ***$p < 0.005$, Student's *t*-test) (**C**) HEK293T cells expressing mock or TRPML3 or TRPML3(C50A) were treated with 100 nM bafilomycin A_1 for 4 h and processed for western blot analysis to assay endogenous LC3 levels. β-actin was used as a loading control.

A

RAB33B	Motif
LIR1	^{34}FxxI^{37}
LIR2	^{104}**YxxV**^{107}
LIR3	^{122}FxxL^{125}
LIR4	^{192}FxxL^{195}

B

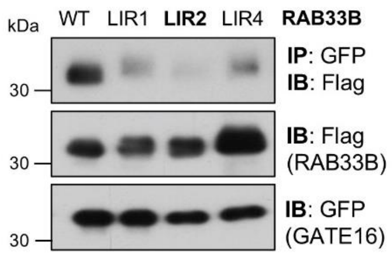

C

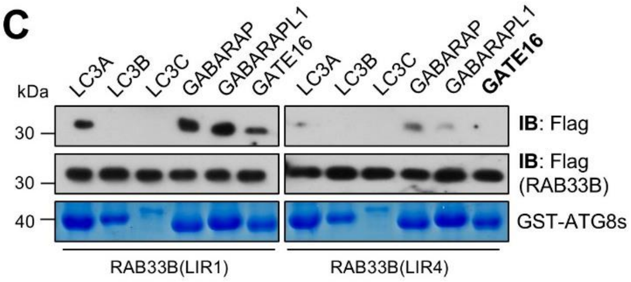

Supplementary Figure 4. Interaction of RAB33B LIR mutants with ATG8 family proteins. (**A**) LIR motifs identified in RAB33B. (**B**) Cell lysates of HEK293T cells expressing Flag-RAB33B LIR mutants with GFP-GATE16 were subjected to immunoprecipitation with anti-GFP antibody and probed with anti-Flag antibody. (**C**) GST-tagged ATG8 homologs coupled to glutathione Sepharose beads were incubated with whole cell lysate from HEK293T cells expressing Flag-RAB33B(LIR1) or Flag-RAB33B(LIR4). Elutes were subjected to SDS-PAGE and immunoblotted with anti-Flag antibody.

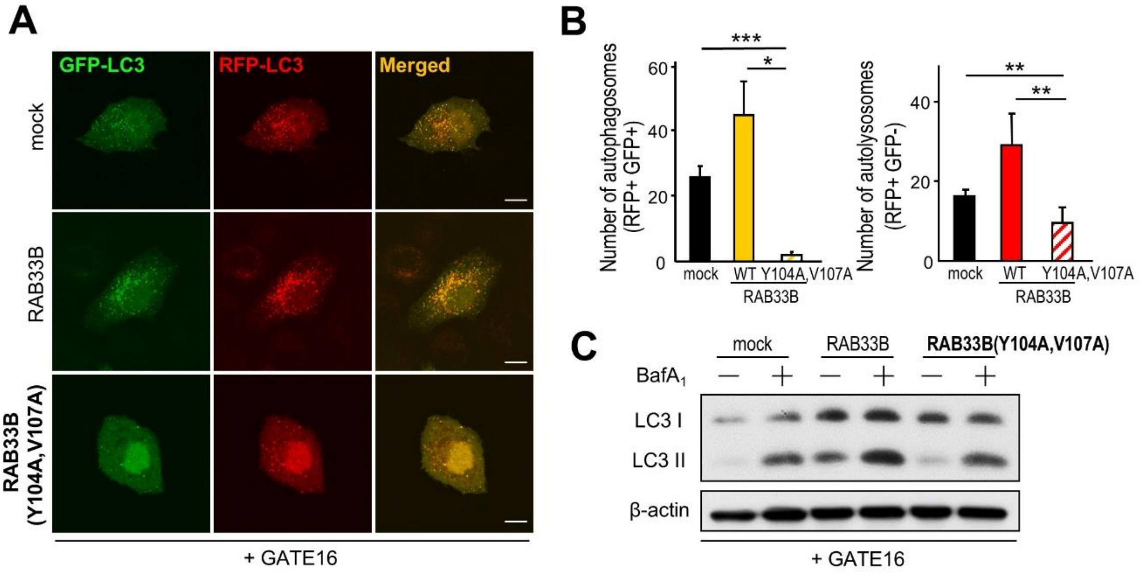

Supplementary Figure 5. Effects of RAB33B and RAB33B(Y104A,V107A) on autophagy flux under fed conditions. (**A**) HeLa cells expressing tfLC3 and Flag-GATE16 with mock, WT, Y104A,V107A RAB33B were analyzed by confocal microscopy. (**B**) The number of autophagosomes and autolysosomes in panel (C) were quantified and presented as mean ± SEM of 3-5 cells (**p* < 0.05, ***p* < 0.01, ****p* < 0.005, Student's *t*-test). (**C**) HEK293T cells expressing mock, RAB33B, RAB33B(Y104A,V107A) were treated with 100 nM bafilomycin A_1 for 2 h and processed for western blot analysis to assay endogenous LC3 levels. β-actin was used as a loading control.
