# Supplementary material for: Two specific interactions of GATE16 with TRPML3 and RAB33B regulate autophagy
Source: Sci Rep. 2025 Aug 25;15:31244. doi: 10.1038/s41598-025-16951-0 (PMC12378212; doi:10.1038/s41598-025-16951-0)

Two specific interactions of GATE16 with TRPML3 and RAB33B regulate autophagy

Jiwoo Park[1,2], Areum Choi[1,2], Jin Kwon[1], Suzi Choi[1], Yun Min Park[1], and Hyun Jin Kim[1,*]

Western blot images

Figure 1

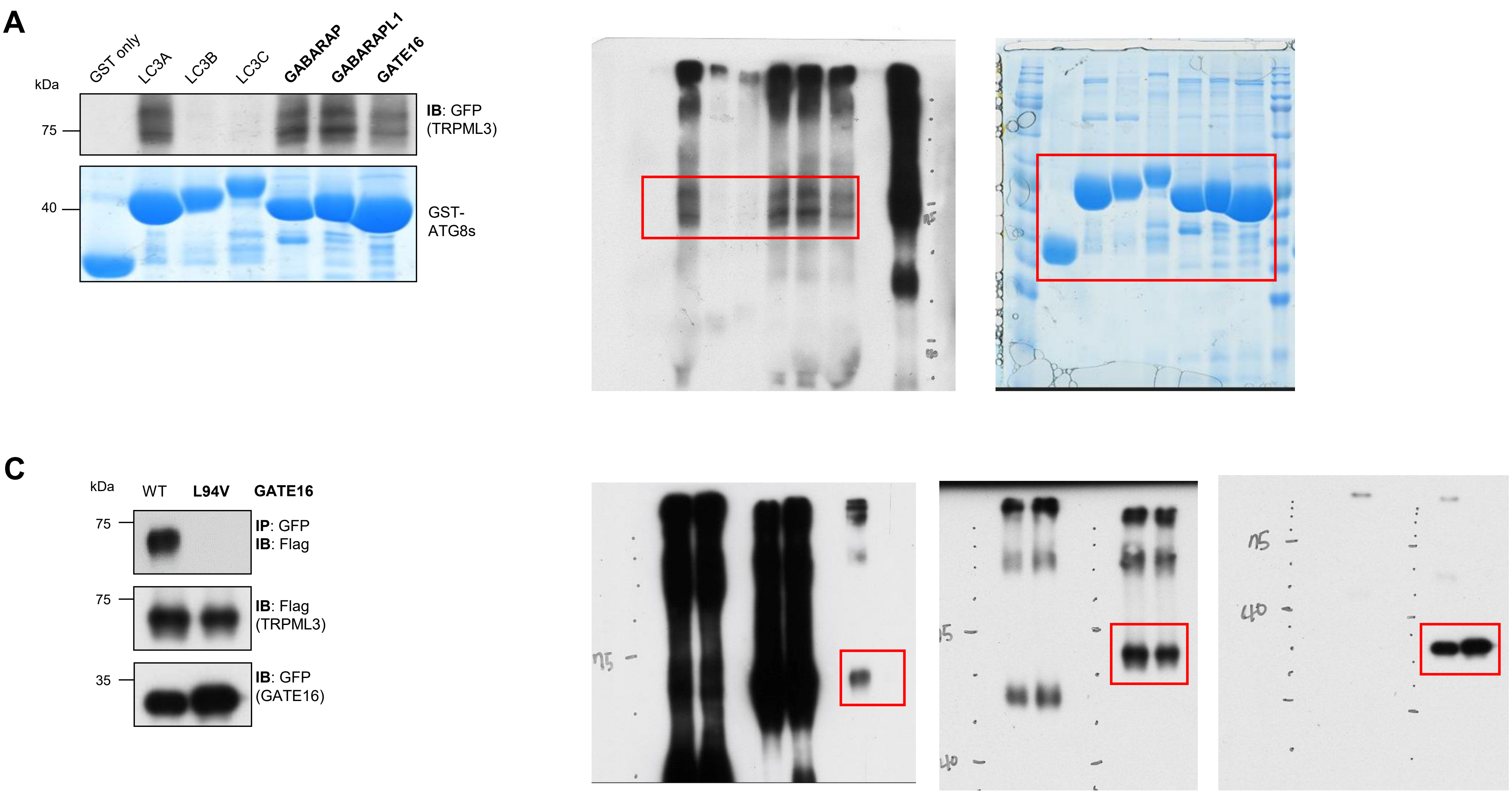
A
kDa
GST only
LC3A
LC3B
LC3C
GABARAP
GABARAPL1
GATE16
75
40
IB: GFP
(TRPML3)
GST-
ATG8s
C
kDa
WT
L94V
GATE16
75
IP: GFP
IB: Flag
75
IB: Flag
(TRPML3)
35
IB: GFP
(GATE16)

Figure 1

D

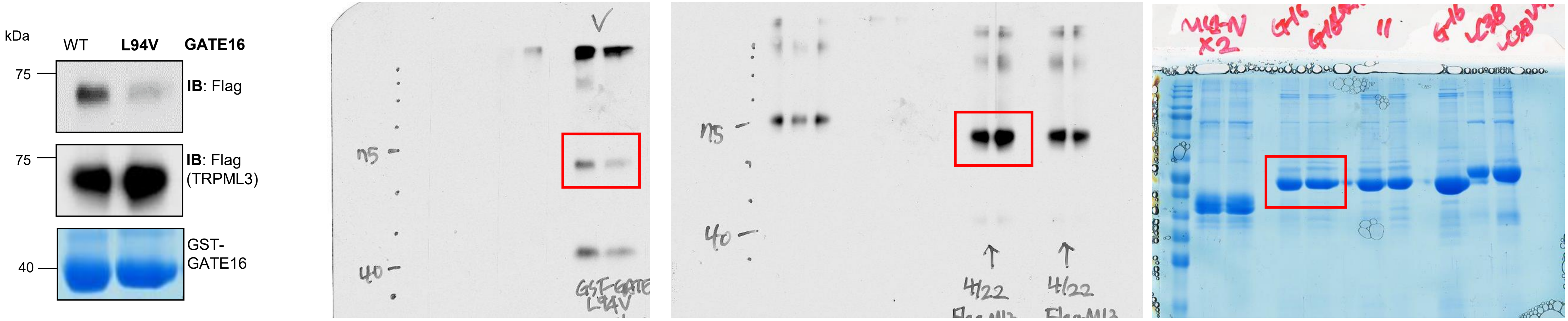
kDa
WT
L94V
GATE16
75
IB: Flag
75
IB: Flag (TRPML3)
40
GST-GATE16

E

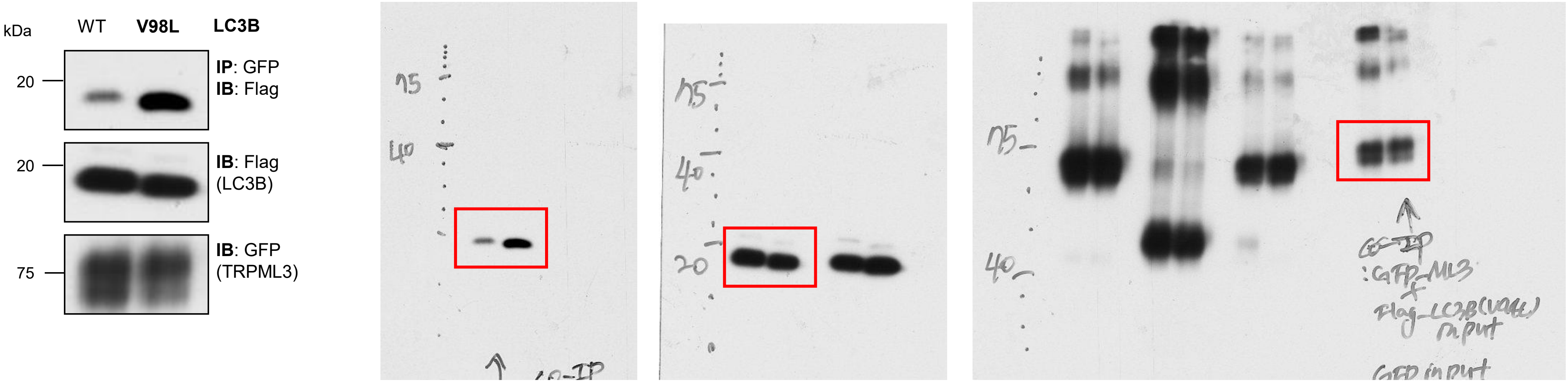
kDa
WT
V98L
LC3B
20
IP: GFP
IB: Flag
20
IB: Flag (LC3B)
75
IB: GFP (TRPML3)

Figure 1

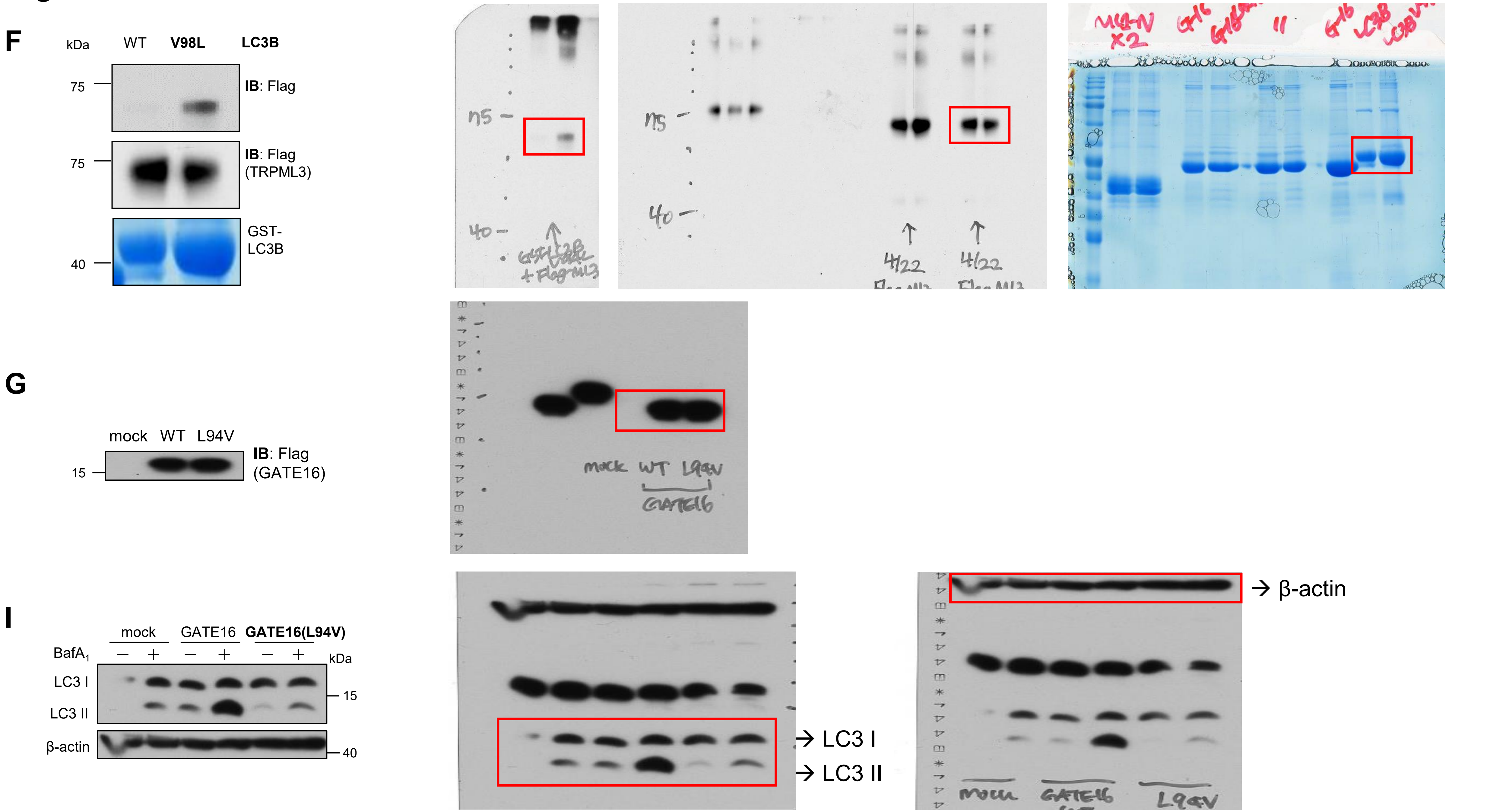
F
kDa
WT
V98L
LC3B
75
IB: Flag
75
IB: Flag (TRPML3)
40
GST-LC3B
G
mock
WT
L94V
15
IB: Flag (GATE16)
I
mock
GATE16
GATE16(L94V)
BafA1
kDa
LC3 I
LC3 II
15
β-actin
40
→ LC3 I
→ LC3 II
→ β-actin

Figure 2

B

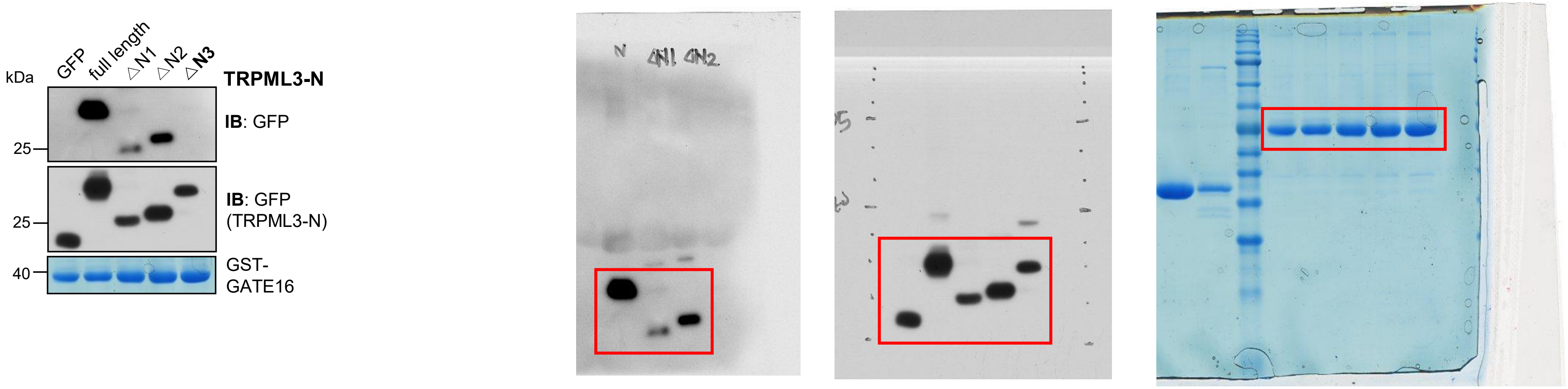
kDa
GFP
full length
△N1
△N2
△N3
TRPML3-N
IB: GFP
25
IB: GFP
(TRPML3-N)
25
40
GST-
GATE16

C

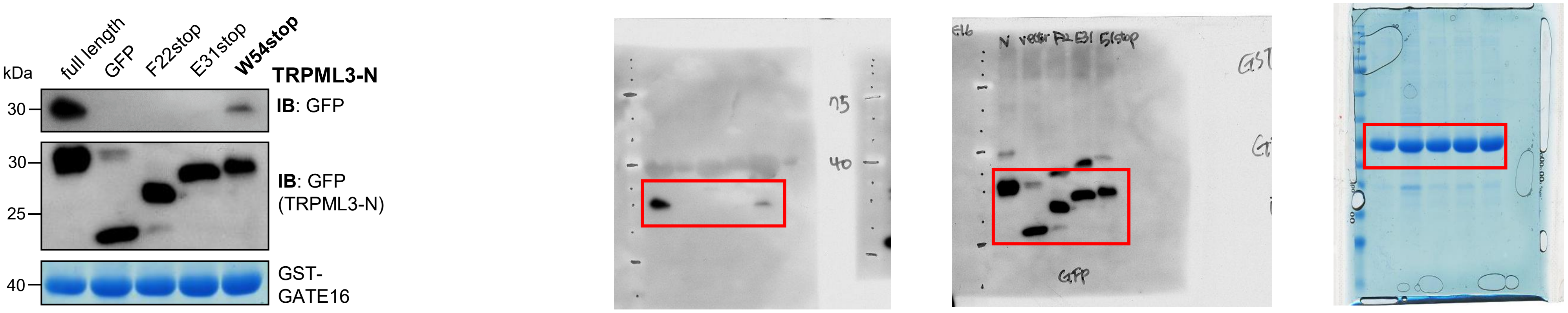
kDa
full length
GFP
F22stop
E31stop
W54stop
TRPML3-N
30
IB: GFP
30
25
IB: GFP
(TRPML3-N)
40
GST-
GATE16

Figure 2

D

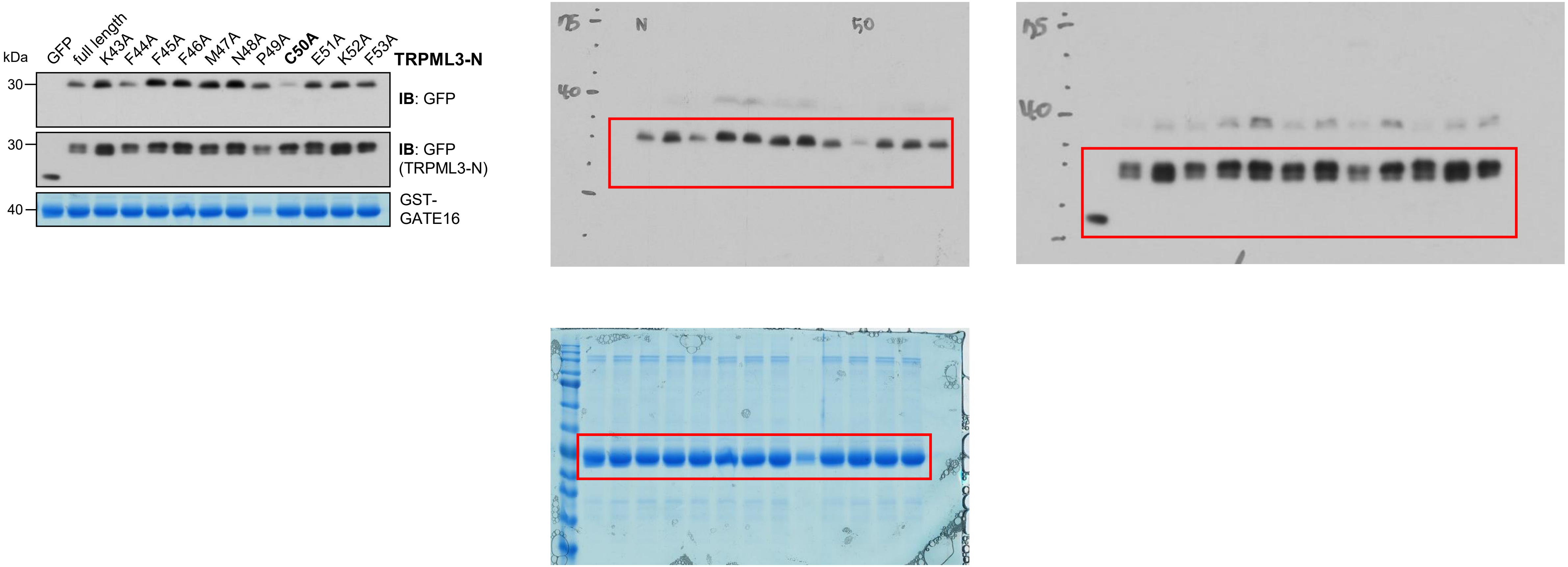

kDa
GFP
full length
K43A
F44A
F45A
F46A
M47A
N48A
P49A
C50A
E51A
K52A
F53A
TRPML3-N
30
IB: GFP
30
IB: GFP (TRPML3-N)
40
GST-GATE16

Figure 2

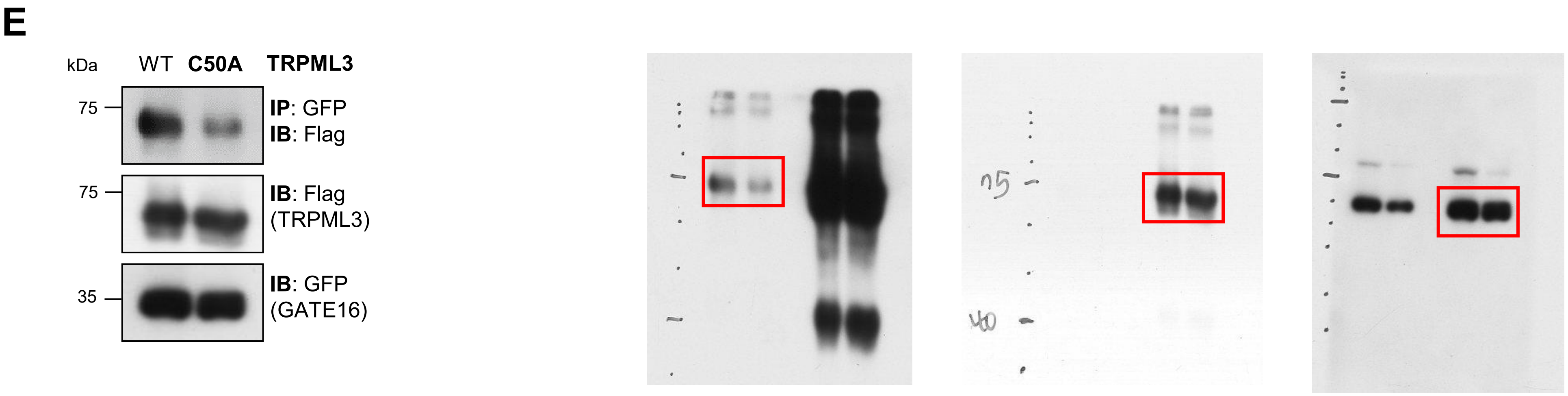

E
kDa
WT
C50A
TRPML3
75
IP: GFP
IB: Flag
75
IB: Flag
(TRPML3)
35
IB: GFP
(GATE16)
75
40

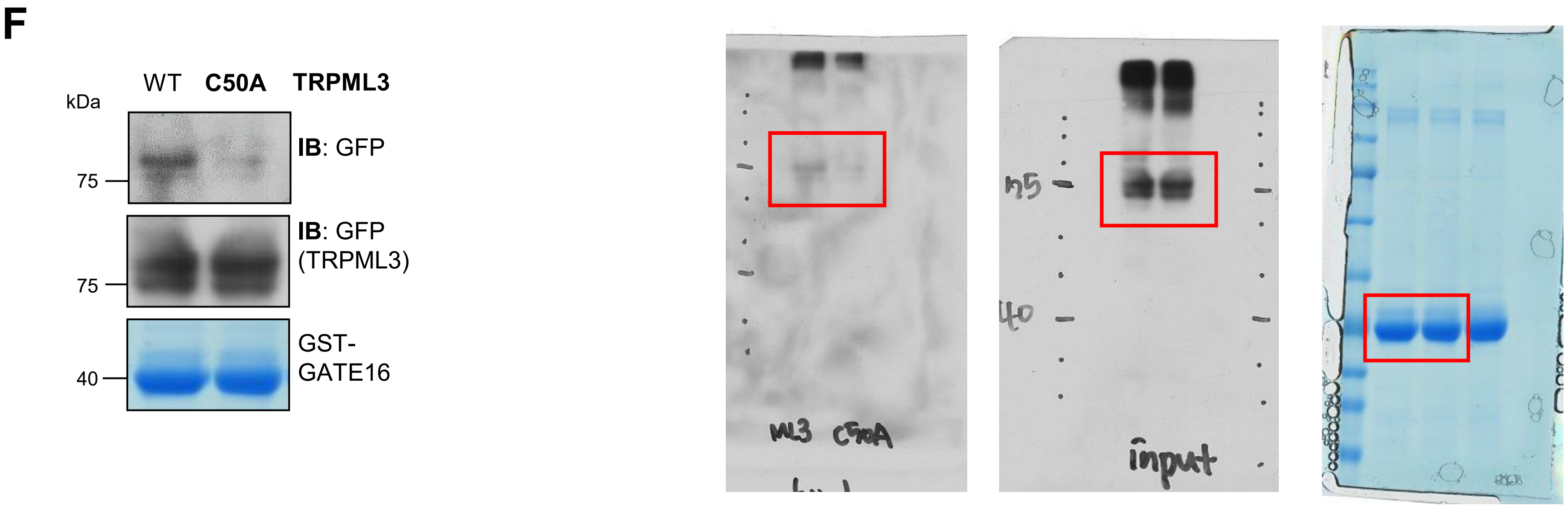

F
WT
C50A
TRPML3
kDa
IB: GFP
75
IB: GFP
(TRPML3)
75
GST-
GATE16
40
75
40
input

Figure 2

G

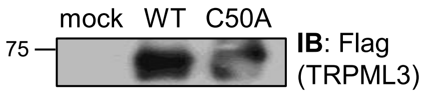

mock
WT
C50A
75
IB: Flag
(TRPML3)

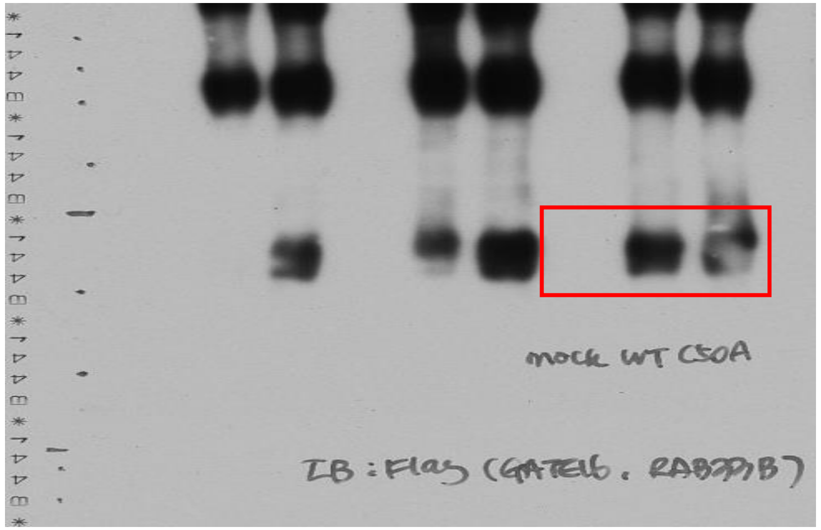

mock WT C50A

I

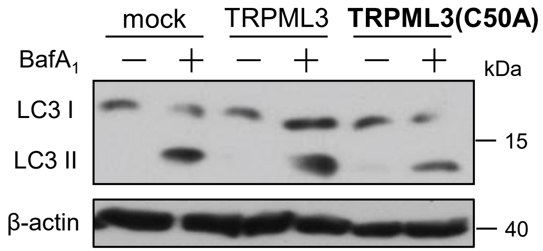

mock
TRPML3
TRPML3(C50A)
BafA1
— + — + — +
kDa
LC3 I
LC3 II
15
β-actin
40

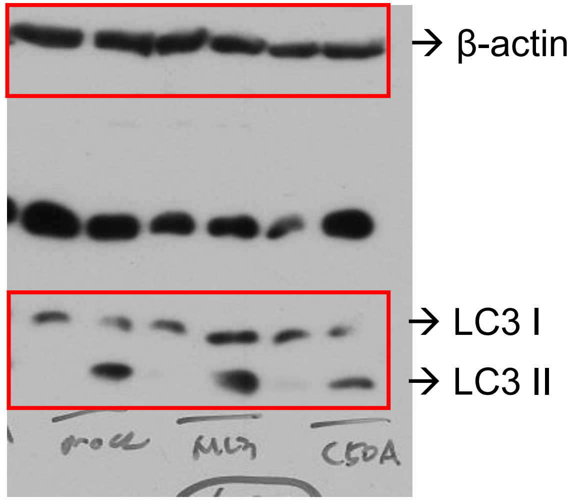

→ β-actin
→ LC3 I
→ LC3 II
C50A

Figure 3

B

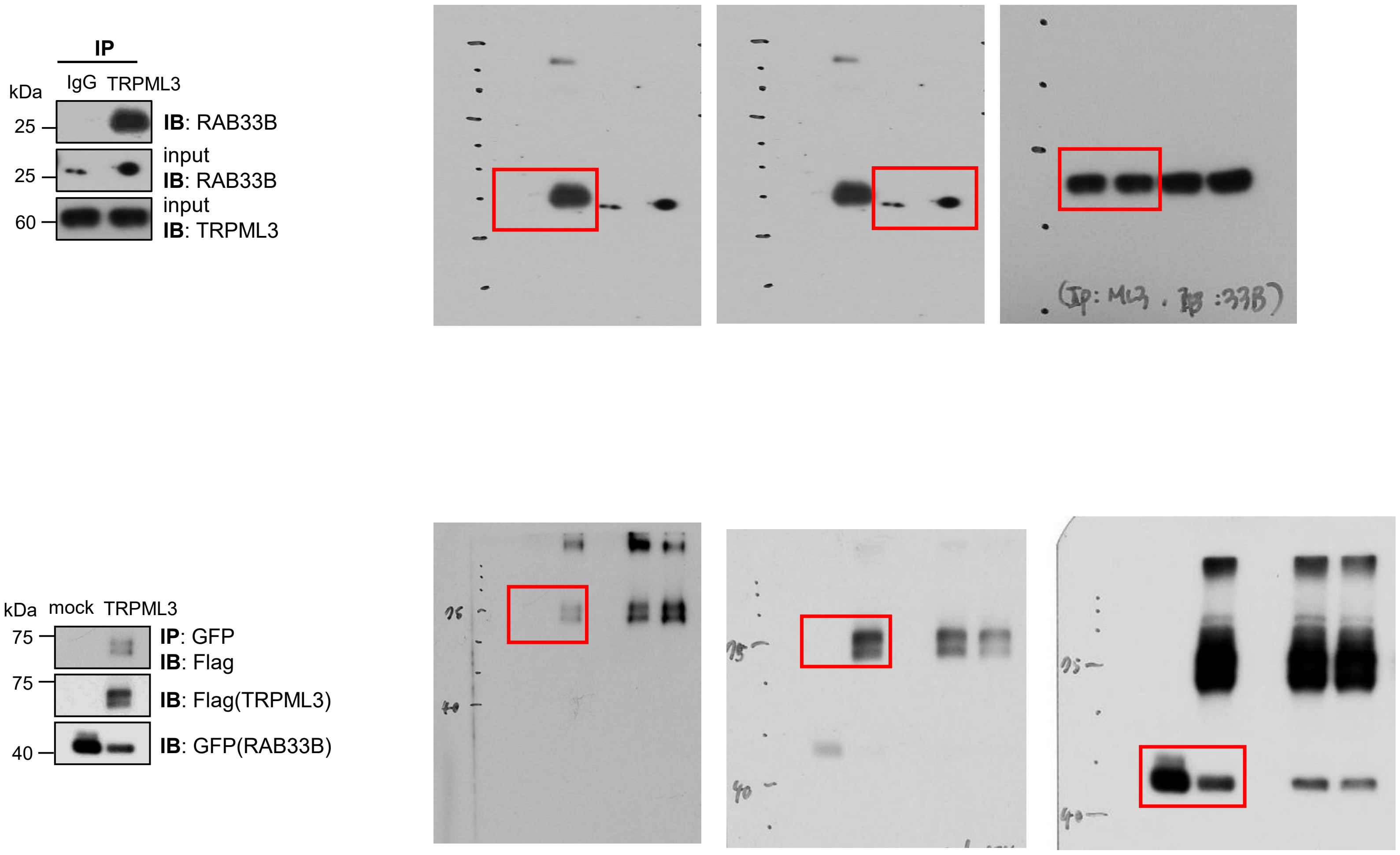
IP
IgG
TRPML3
kDa
25
IB: RAB33B
input
IB: RAB33B
25
input
IB: TRPML3
60
kDa
mock
TRPML3
75
IP: GFP
IB: Flag
75
IB: Flag(TRPML3)
40
IB: GFP(RAB33B)

Figure 3

C

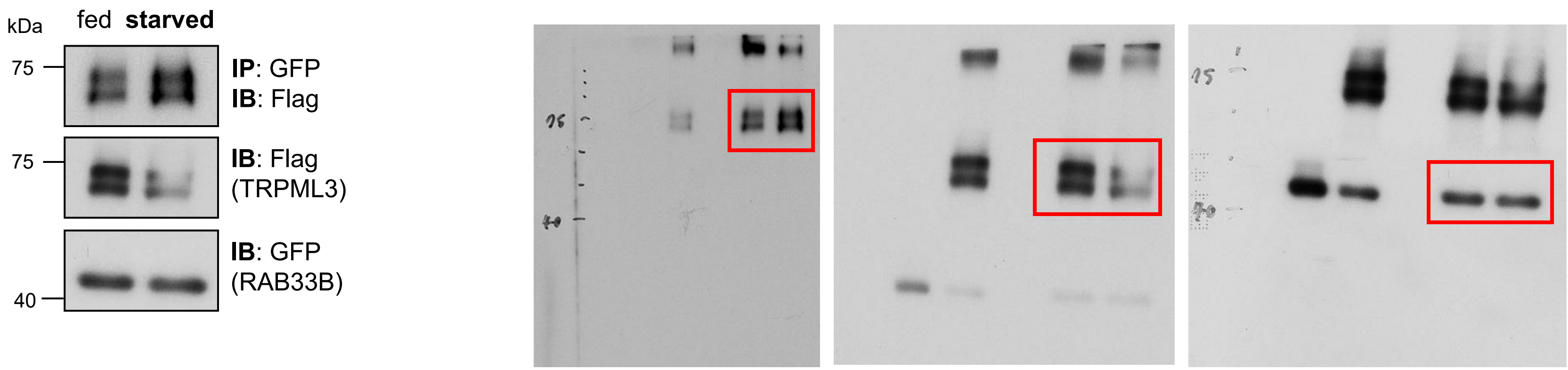
kDa
fed
starved
75
IP: GFP
IB: Flag
75
IB: Flag
(TRPML3)
IB: GFP
(RAB33B)
40

D

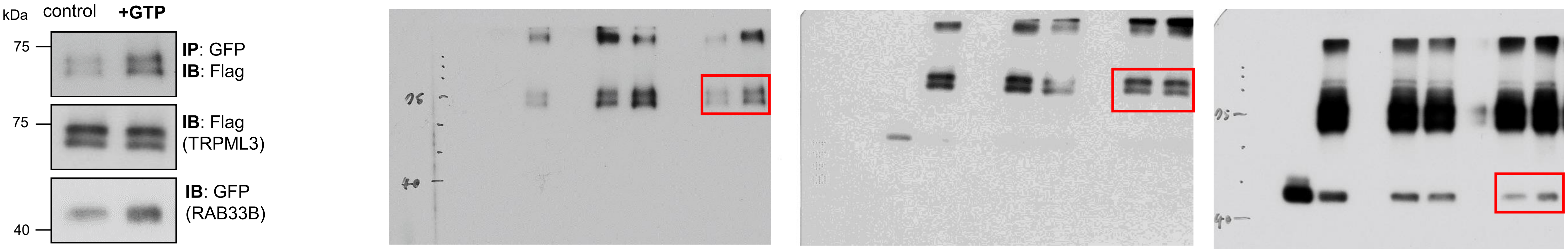
kDa
control
+GTP
75
IP: GFP
IB: Flag
75
IB: Flag
(TRPML3)
IB: GFP
(RAB33B)
40

Figure 3

E

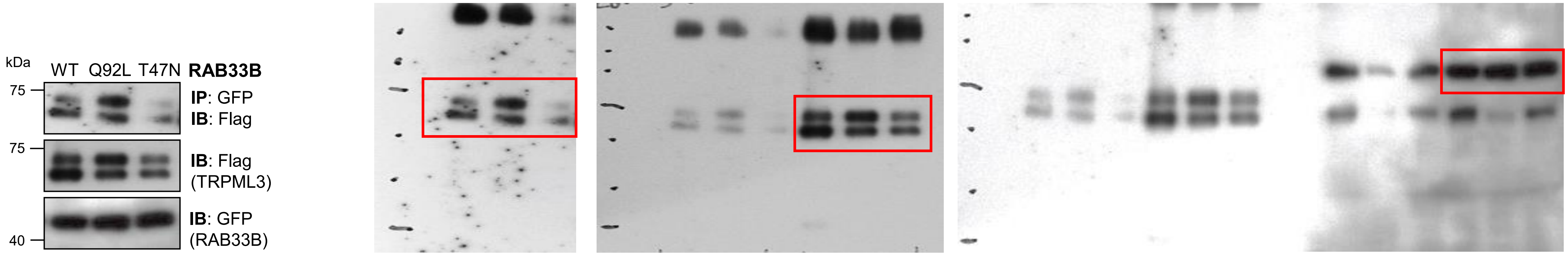

kDa
WT
Q92L
T47N
RAB33B
75
IP: GFP
IB: Flag
75
IB: Flag
(TRPML3)
IB: GFP
40
(RAB33B)

F

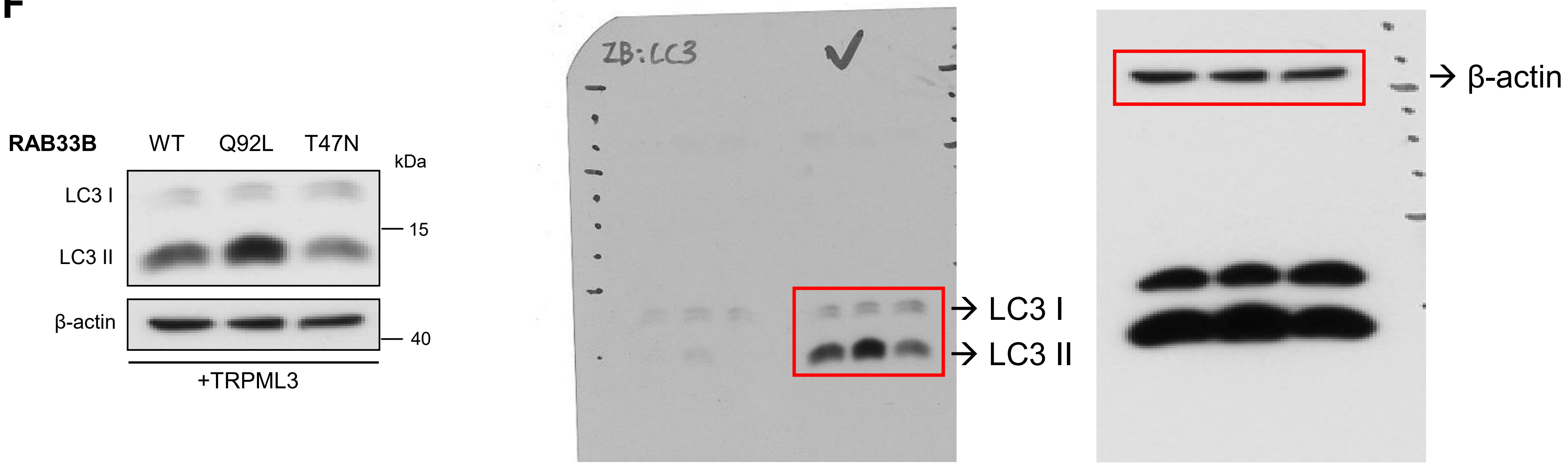

ZB: LC3
→ LC3 I
→ LC3 II
→ β-actin
RAB33B
WT
Q92L
T47N
kDa
LC3 I
15
LC3 II
β-actin
40
+TRPML3

Figure 4

A

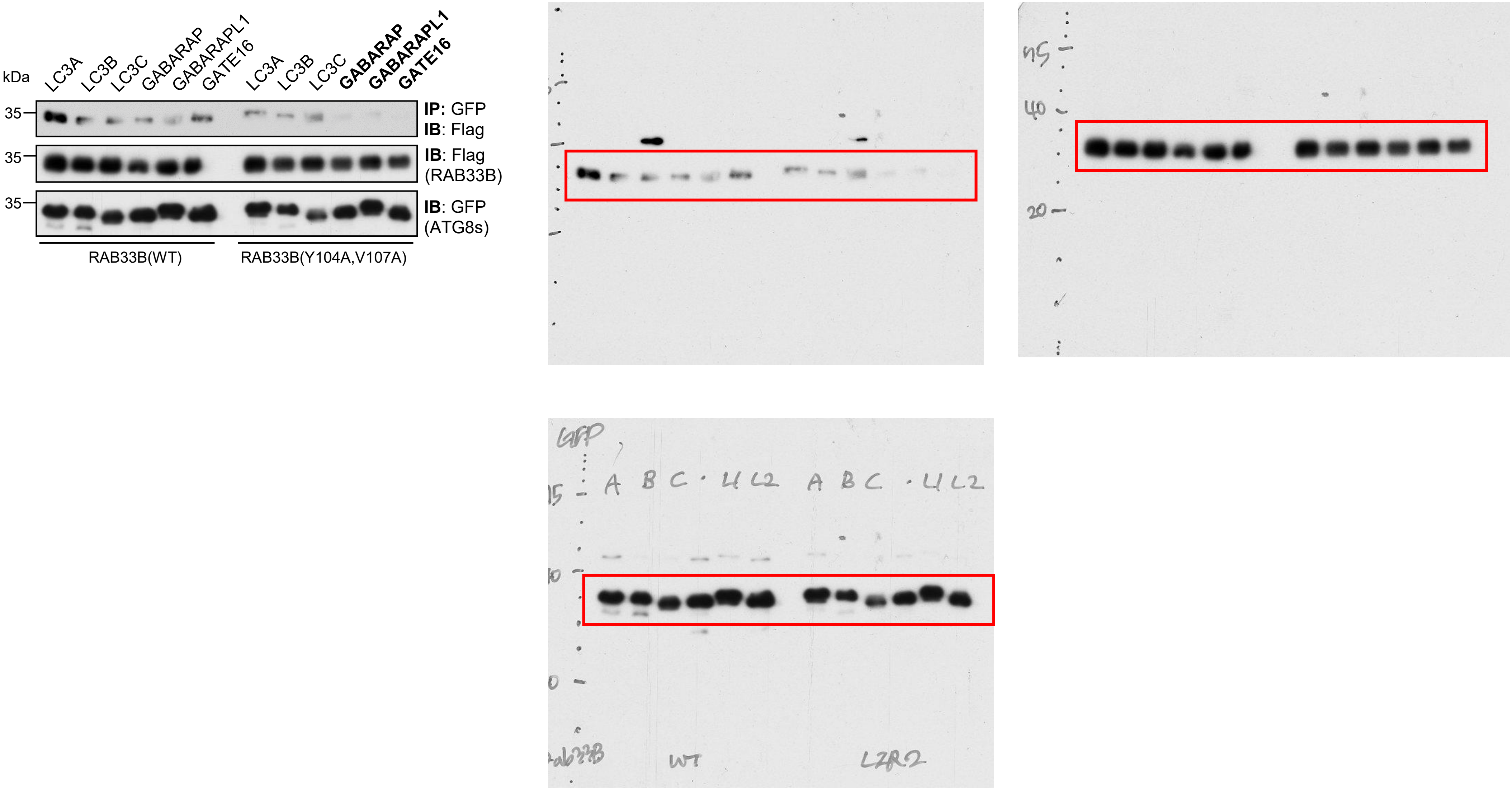

kDa
LC3A
LC3B
LC3C
GABARAP
GABARAPL1
GATE16
LC3A
LC3B
LC3C
GABARAP
GABARAPL1
GATE16
35
35
35
IP: GFP
IB: Flag
IB: Flag
(RAB33B)
IB: GFP
(ATG8s)
RAB33B(WT)
RAB33B(Y104A,V107A)

Figure 4

B

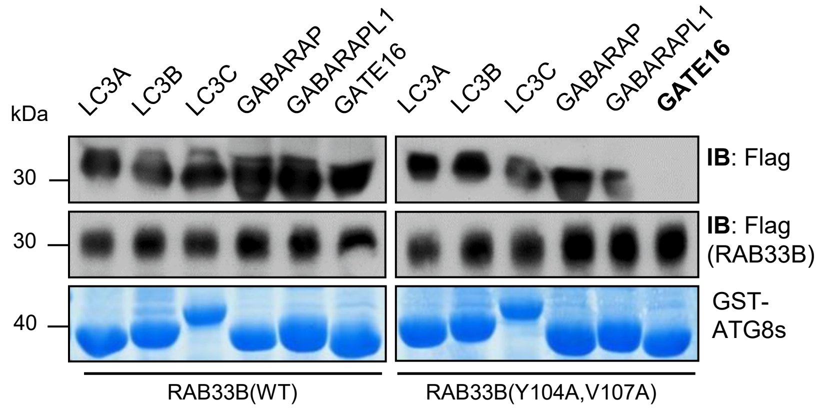

LC3A
LC3B
LC3C
GABARAP
GABARAPL1
GATE16
LC3A
LC3B
LC3C
GABARAP
GABARAPL1
GATE16
kDa
30
30
40
IB: Flag
IB: Flag (RAB33B)
GST-ATG8s
RAB33B(WT)
RAB33B(Y104A,V107A)

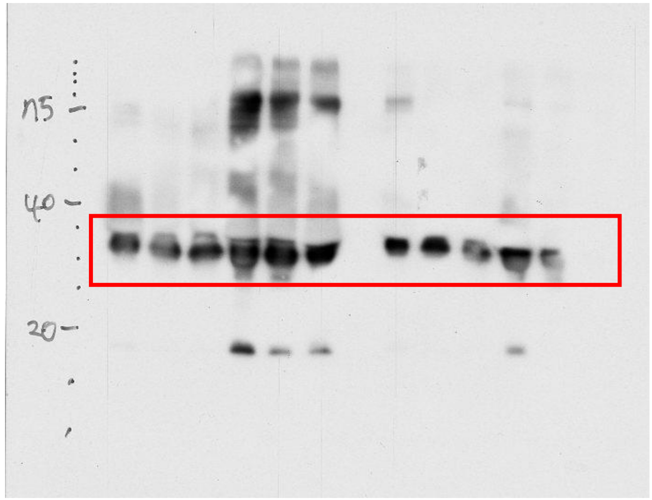

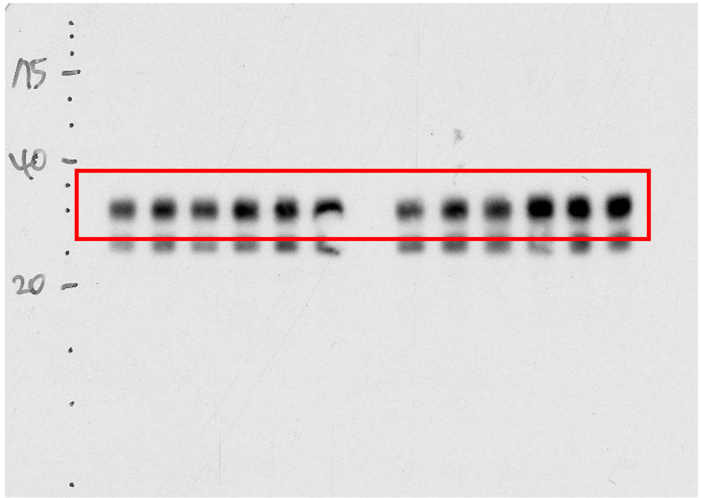

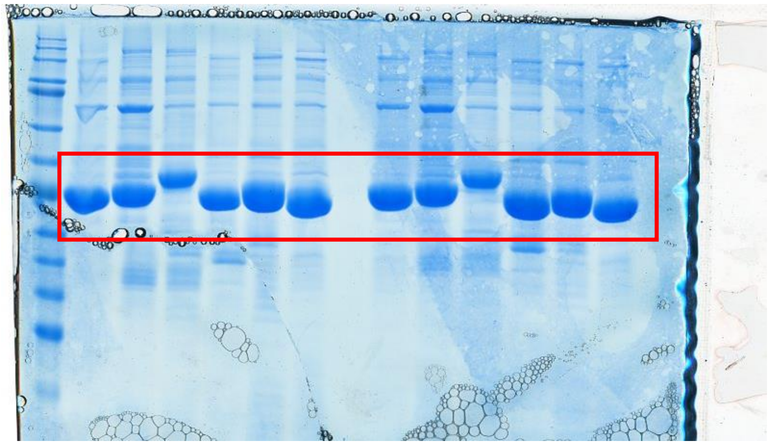

Figure 4

C

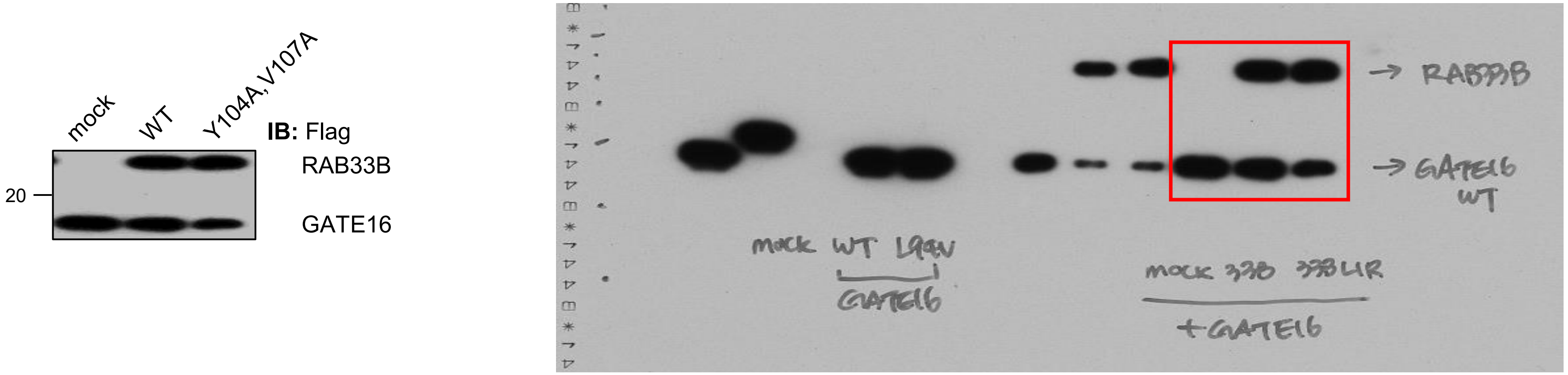
mock
WT
Y104A,V107A
IB: Flag
RAB33B
20
GATE16

E

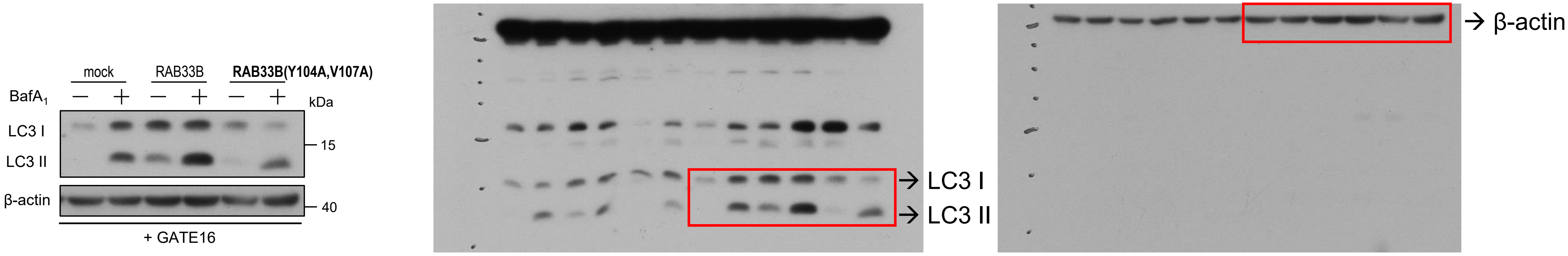
mock
RAB33B
RAB33B(Y104A,V107A)
BafA1
kDa
LC3 I
LC3 II
15
β-actin
40
+ GATE16
LC3 I
LC3 II
β-actin

Figure 5

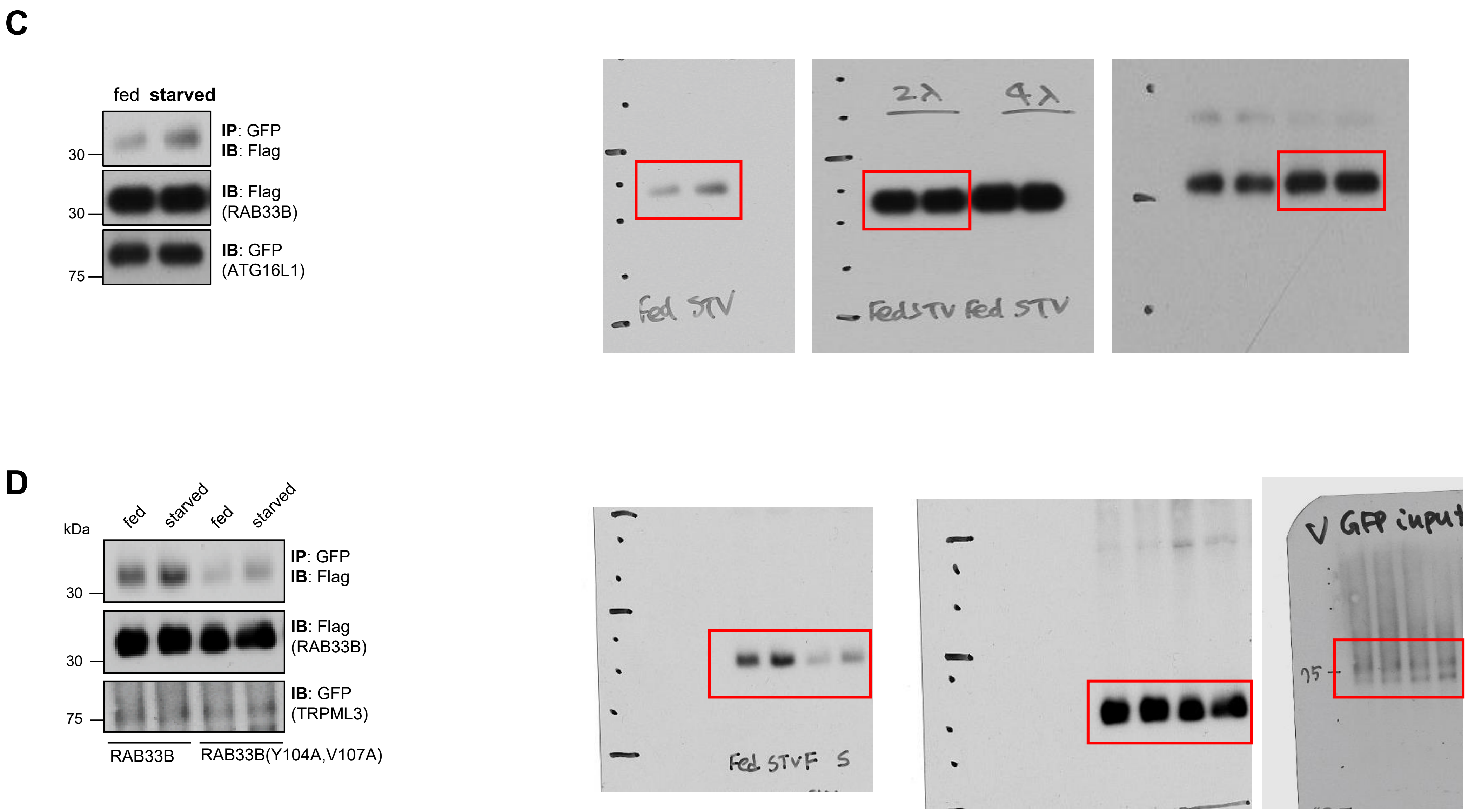
C
fed
starved
30
30
75
IP: GFP
IB: Flag
IB: Flag
(RAB33B)
IB: GFP
(ATG16L1)
fed STV
2λ
4λ
Fed STV Fed STV
D
kDa
fed
starved
fed
starved
30
30
75
IP: GFP
IB: Flag
IB: Flag
(RAB33B)
IB: GFP
(TRPML3)
RAB33B
RAB33B(Y104A,V107A)
Fed STV F S
V GFP input
75

Supplementary Figure 2

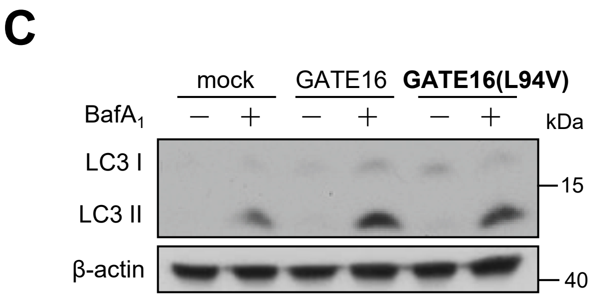

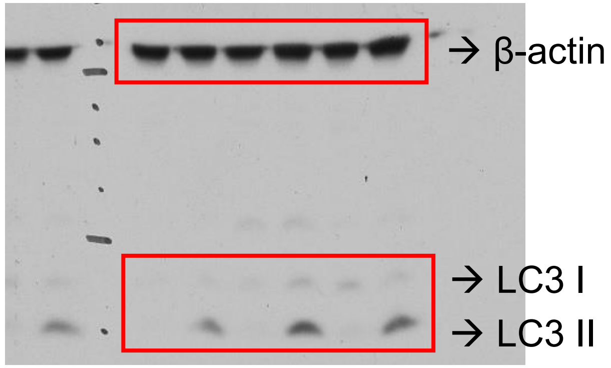

Supplementary Figure 3

C

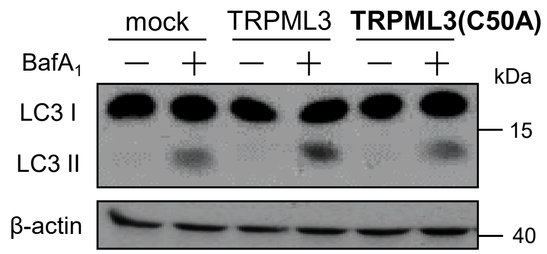

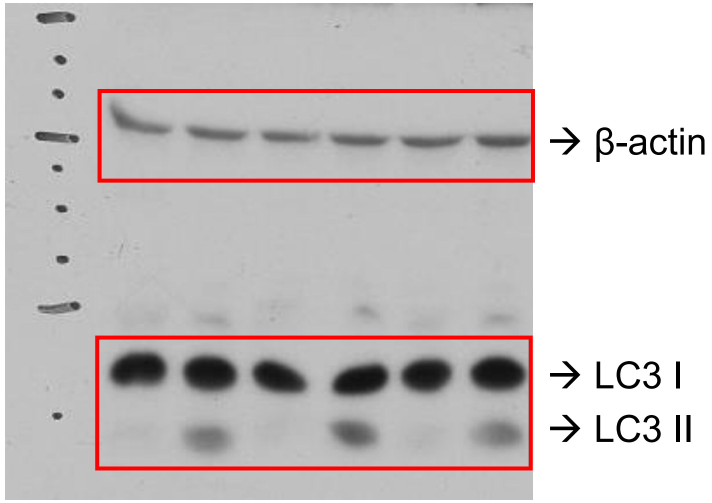

Supplementary Figure 4

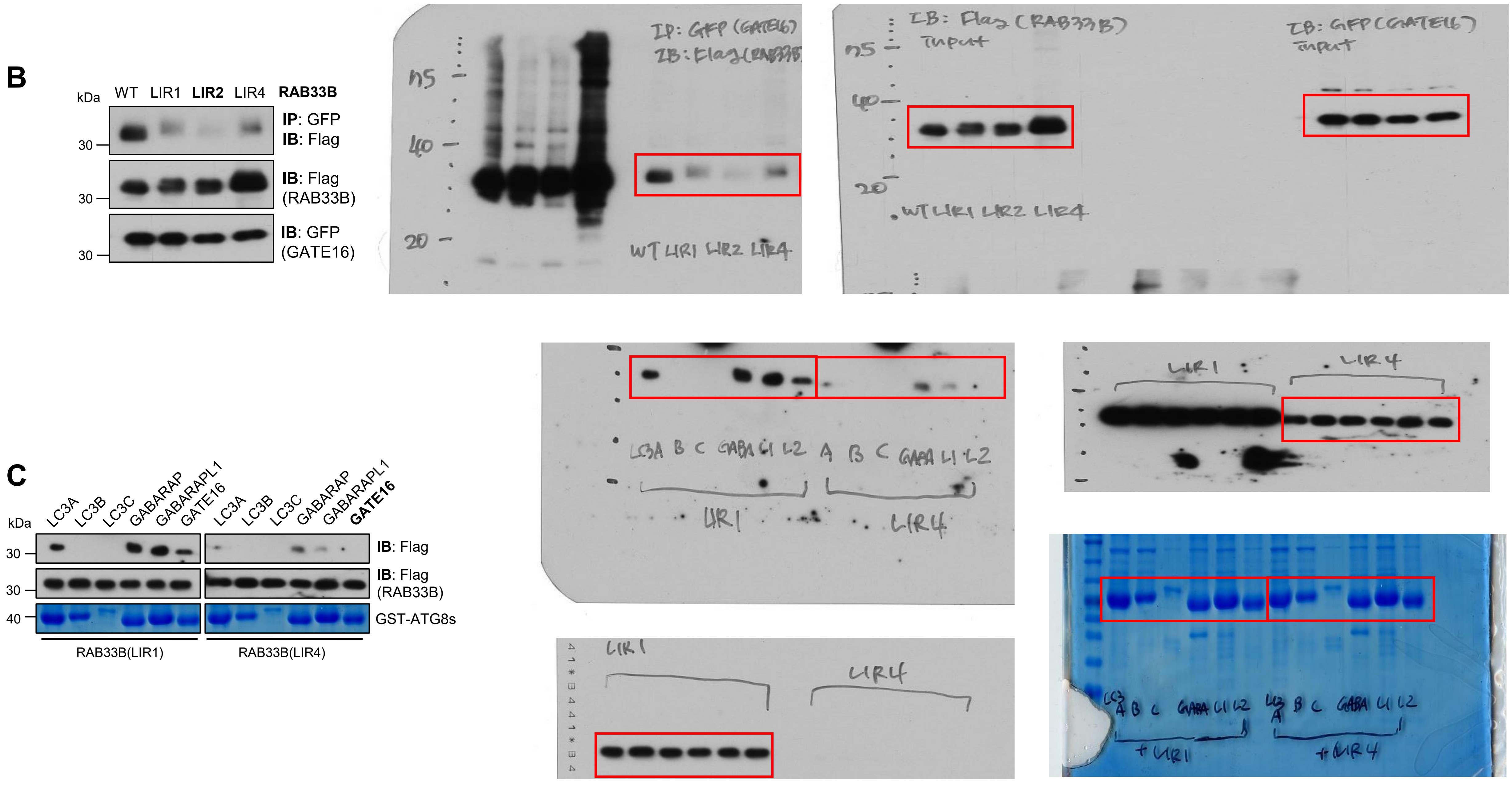

Supplementary Figure 5

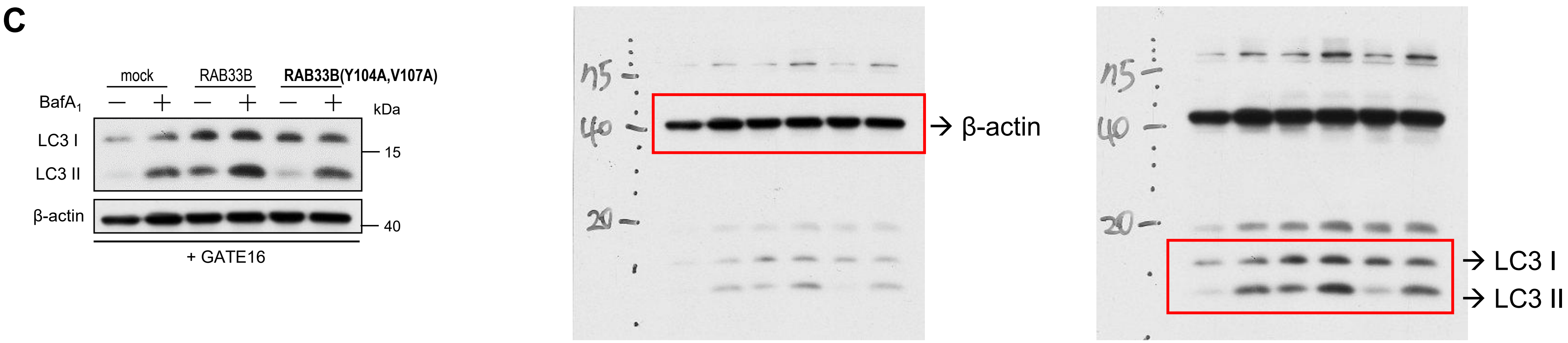

Supplement: Supplementary file 2 — Supplementary Material 2 [file 41598_2025_16951_MOESM2_ESM.pdf]
